# Adaptive laboratory evolution of maltose utilization in wild Irish isolates of *Saccharomyces eubayanus*

**DOI:** 10.1093/femsyr/foag036

**Published:** 2026-08-08

**Authors:** Sean Bergin, Jillian Scully, Conor Hession, Juliane da Silveira, Matthieu Osborne, Calum Nolan, Róisín O’Neill, Hans-Georg Eckhardt, Lisa Lombardi, Kenneth H Wolfe, Geraldine Butler

**Affiliations:** School of Biomolecular and Biomedical Science, Conway Institute, University College Dublin, Belfield, Dublin 4, D04 V1W8, Ireland; School of Biomolecular and Biomedical Science, Conway Institute, University College Dublin, Belfield, Dublin 4, D04 V1W8, Ireland; School of Biomolecular and Biomedical Science, Conway Institute, University College Dublin, Belfield, Dublin 4, D04 V1W8, Ireland; School of Biomolecular and Biomedical Science, Conway Institute, University College Dublin, Belfield, Dublin 4, D04 V1W8, Ireland; School of Medicine, Conway Institute, University College Dublin, Belfield, Dublin 4, D04 V1W8, Ireland; School of Biomolecular and Biomedical Science, Conway Institute, University College Dublin, Belfield, Dublin 4, D04 V1W8, Ireland; School of Biomolecular and Biomedical Science, Conway Institute, University College Dublin, Belfield, Dublin 4, D04 V1W8, Ireland; School of Chemistry, University College Dublin, Belfield, Dublin 4, D04 V1W8, Ireland; Department of Biology, University of Pisa, 56126 Pisa, Italy; School of Medicine, Conway Institute, University College Dublin, Belfield, Dublin 4, D04 V1W8, Ireland; School of Biomolecular and Biomedical Science, Conway Institute, University College Dublin, Belfield, Dublin 4, D04 V1W8, Ireland

**Keywords:** yeast, maltose, *Saccharomyces eubayanus*, evolution

## Abstract

The first European isolates of *Saccharomyces eubayanus* were discovered in Ireland in 2022 and belong to the same clade as the *S. eubayanus* parent of the hybrid lager yeast *Saccharomyces pastorianus*. Here, we report the isolation of 10 additional Irish strains and we explore maltose metabolism. Maltose metabolism genes are clustered in subtelomeric *MAL* loci including maltase (*MALS*), maltose transporter (*MALT*), and regulator (*MALR*). Despite having intact *MAL* loci, the Irish strains do not grow on maltose. Two Irish *S. eubayanus* strains (UCD650 and UCD926) were passaged in medium containing maltose as the sole carbon source until they acquired the ability to metabolize maltose. The UCD650-derived lineage acquired an I243N substitution in MalR, and a 30-kb duplication of the *MAL* locus. The UCD926 parent has a similar *MAL* locus duplication and its evolved lineage acquired a W307L substitution in MalR. Expression of *MALT* and *MALS* was increased in the evolved isolates. Introducing the W307L mutation into UCD926 by CRISPR-Cas9 editing fully recapitulates the maltose utilization phenotype. The I243N variant alone has little effect on expression of maltose genes. We find that maltose utilization is influenced both by the copy number of *MAL* genes and by gain-of-function mutations in MalR.

## Teaser

In adaptive laboratory evolution, Irish *Saccharomyces eubayanus* strains acquired the ability to grow on maltose mediated by duplication of the entire *MAL* locus and mutations in the regulatory gene *MALR*.

## Introduction

Beer production is an ancient practice that has continued relevance in global human culture (Samuel 1996, Jennings et al. 2005, Raihofer et al. 2022). In 2024 alone, it is estimated that 188 billion litres of beer were produced (Barth-HaasGroup 2024). Most beer production is possible because of the unintended domestication of two yeast species that can ferment sugars present in malted grains into alcohol (Legras et al. 2007, Gallone et al. 2016). One species, *Saccharomyces cerevisiae*, has been associated with the production of ale-type beer since prehistory, and is also used in wine production, bread-making, and more (Legras et al. 2007). The other species, *Saccharomyces pastorianus*, is a hybrid, allopolyploid yeast that has been used for hundreds of years in the production of lager beer (Raihofer et al. 2022), which has come to dominate the beer markets (Howard 2014). This hybrid yeast has only ever been found in brewing conditions, leading to the assumption that the original hybridization event occurred in a brewhouse (Hutzler et al. 2023). While it has long been known that *S. cerevisiae* was one parent to the hybrid (Dunn and Sherlock 2008), the other parent remained a mystery until 2011 when *Saccharomyces eubayanus* was identified in Argentina (Libkind et al. 2011). Since its initial discovery, *S. eubayanus* has been isolated from North America (Peris et al. 2014, Peris et al. 2016, Langdon et al. 2020), Tibet (Bing et al. 2014), New Zealand (Gayevskiy and Goddard 2016), Ireland (Bergin et al. 2022), and many more times in South America (Eizaguirre et al. 2018, Nespolo et al. 2020, Villarreal et al. 2026).

Distinct from *S. pastorianus, S. eubayanus* has itself been used for alcohol production both in traditional practices in South America and recently at commercial scale. *Saccharomyces eubayanus* was the sole *Saccharomyces* species present in a traditional Andean apple chicha fermentation where it dominated in the late stages of fermentation (Gonzalez‐Flores et al. 2023). It likely originated as a spontaneous fermentation due to association with local feral apple trees (Gonzalez‐Flores et al. 2023). There has also been archaeological evidence for pre-Hispanic fermentation using *S. eubayanus* from strains isolated from earthenware pots dated 1000–1300 CE (Pérez et al. 2025). Following its initial identification in 2011, *S. eubayanus* strains from across the globe have been exploited for lager production, particularly by Heineken’s limited Wild Lager Exploration series (Giorgetti et al. 2025).

During beer brewing, various biosynthetic pathways convert carbohydrates available in the complex medium wort into ethanol, higher alcohols, and esters that provide aroma to the beer (Stewart 2017). Wort contains several carbohydrates including sucrose, fructose, and glucose, but the oligosaccharides maltose and maltotriose comprise the majority of available sugars (Otter and Taylor 1967, He et al. 2014). In yeast, simple sugars are initially preferred due to glucose-sensitive carbon catabolite repression of the genes targeting more complex sugars, until depletion of glucose followed by de-repression allows for a diauxic shift towards the use of more complex sugars (Gancedo 1993, Gancedo 1998).

Wild Patagonian isolates of *S. eubayanus* have a remarkable variation in capacity for maltose metabolism (Mardones et al. 2020). The *S. eubayanus* type strain, CBS 12357^T^, typically shows fast growth on maltose medium (Gibson et al. 2013) and a strong ability to convert maltose to alcohol (Eizaguirre et al. 2018, Mardones et al. 2020), whereas other Patagonian strains show slower growth and incomplete maltose consumption at the end of fermentation (Molinet et al. 2022). In particular, the strain QC18 displays low fermentation efficiency and poor consumption of maltose (Molinet et al. 2022). Intriguingly, QC18 was isolated from exotic *Quercus robur* in Argentina, whereas most Patagonian isolates that can ferment maltose were associated with native *Nothofagus* species (Eizaguirre et al. 2018, Molinet et al. 2022). Outside of Patagonia, Tibetan strains isolated from rotten wood and Irish strains isolated from soil are unable to grow on maltose as a sole carbon source (Brouwers et al. 2019, Bergin et al. 2022). Villareal et al. (2026) have suggested that there is a selective pressure for maintaining maltose utilization when associated with *Nothofagus* species that is relaxed in other niches, leading to loss of maltose utilization capability (Villarreal et al. 2026). Indeed, Villarreal et al. (2026) have shown that the bark of non-*Nothofagus* hosts of *S. eubayanus* contains less maltose than *Nothofagus* species.

The ability to metabolize maltose in *Saccharomyces* species is regulated by the *MAL* loci (Michels and Needleman 1983, Charron et al. 1986, Naumov et al. 1994). Each *MAL* locus contains genes encoding a transcriptional activator MalR, a maltose permease MalT, and an alpha-glucosidase (maltase) MalS that breaks maltose down into constituent glucose molecules (Charron et al. 1986). In the presence of maltose, MalR upregulates expression of *MALT* and *MALS* by binding to specific activator binding sites in the bidirectional promoter shared by both genes (Novak et al. 2004). In *S. cerevisiae, MAL* loci are typically located in subtelomeric regions and can vary in copy number and location between strains (Brown et al. 2010). It is suggested that genes localized to subtelomeres, such as the *MAL* genes, are subject to higher rates of recombination, and so are more readily duplicated following selective pressure (Brown et al. 2010). Copy number variation has been linked with different capabilities of maltose metabolism, particularly in domesticated strains (Duval et al. 2010, Gallone et al. 2016). To account for multiple copies the typical naming convention used in *S. cerevisiae* is *MALx1* for the transporter, *MALx2* for the maltase, and *MALx3* for the regulator, where the ‘x’ is a number referring to the specific *MAL* locus, e.g. *MAL32* is the maltase found at the *MAL3* locus. However, in this manuscript we show that the phylogenetic relationships among the *MAL* genes of *S. cerevisiae* and *S. eubayanus* are more complex than simple 1:1 orthology, so for *S. eubayanus* we instead use a nomenclature system based on the names *MALR, MALS* and *MALT* with superscripts indicating their chromosomal location.

The *S. eubayanus* type strain CBS 12357^T^ has two complete copies of the *MAL* locus on chromosomes V and XVI, and in addition has copies of some *MAL* genes at two other positions. There is a single *MALT* transporter gene on chromosome II, and a *MALT* gene flanked by two copies of the *MALR* regulator on chromosome XIII. Interestingly, the *MALT* copies present in the complete *MAL* loci have higher expression levels in maltose-rich conditions than the others (Brickwedde et al. 2018). Some Holarctic clade strains ranging from North America, Europe, and the Himalayas are incapable of metabolizing maltose despite also having a full complement of *MAL* genes (Brouwers et al. 2019, Bergin et al. 2022, Villarreal et al. 2026). This is likely due to defects in upstream regulation as introduction of a heterologous *S. cerevisiae MAL13* regulator into Himalayan strain CDFM21L.1 enables growth on maltose (Brouwers et al. 2019). Similarly, introduction of the *MALR* gene from chromosome V of CBS 12357^T^ (also referred to as *MAL33*) is sufficient to allow growth on maltose of Irish and North American strains that cannot normally metabolize the sugar (Villarreal et al. 2026). Similarly, overexpression of endogenous *MAL33* improved maltose growth in maltose-deficient Patagonian strain QC18 (Quintrel et al. 2026).

We previously described the discovery of the first European isolates of *S. eubayanus*, isolated from soil on the campus of University College Dublin in Ireland (Bergin et al. 2022). Here we describe ten additional Irish strains isolated from seven sites across Ireland. Most of these contain at least one intact *MAL* locus, and yet they all display poor growth on maltose as a sole carbon source. Adaptive laboratory evolution of two strains (UCD650 and UCD926) resulted in acquisition of ability to metabolize maltose. Both strains acquired mutations in *MALR* which we hypothesize are gain-of-function mutations that allow for increased expression of the regulatory targets of *MALR*. Additionally, during laboratory evolution UCD650 underwent a duplication of the left arm of chromosome XVI containing the *MAL* locus. A similar duplication is present naturally in the original isolate of UCD926. Quantitative RT-PCR shows that expression of *MALS* and *MALT* is increased in the evolved strains in maltose growth conditions as compared to their wild-type progenitors. Introduction of the *MALR* mutations by CRISPR-Cas9 editing into a wild-type parent containing a *MAL* duplication restores the maltose growth phenotype and increases the expression of *MALS* and *MALT*.

## Results

### Isolation and genome sequencing of additional Irish *S. eubayanus* strains

**Figure 1:**
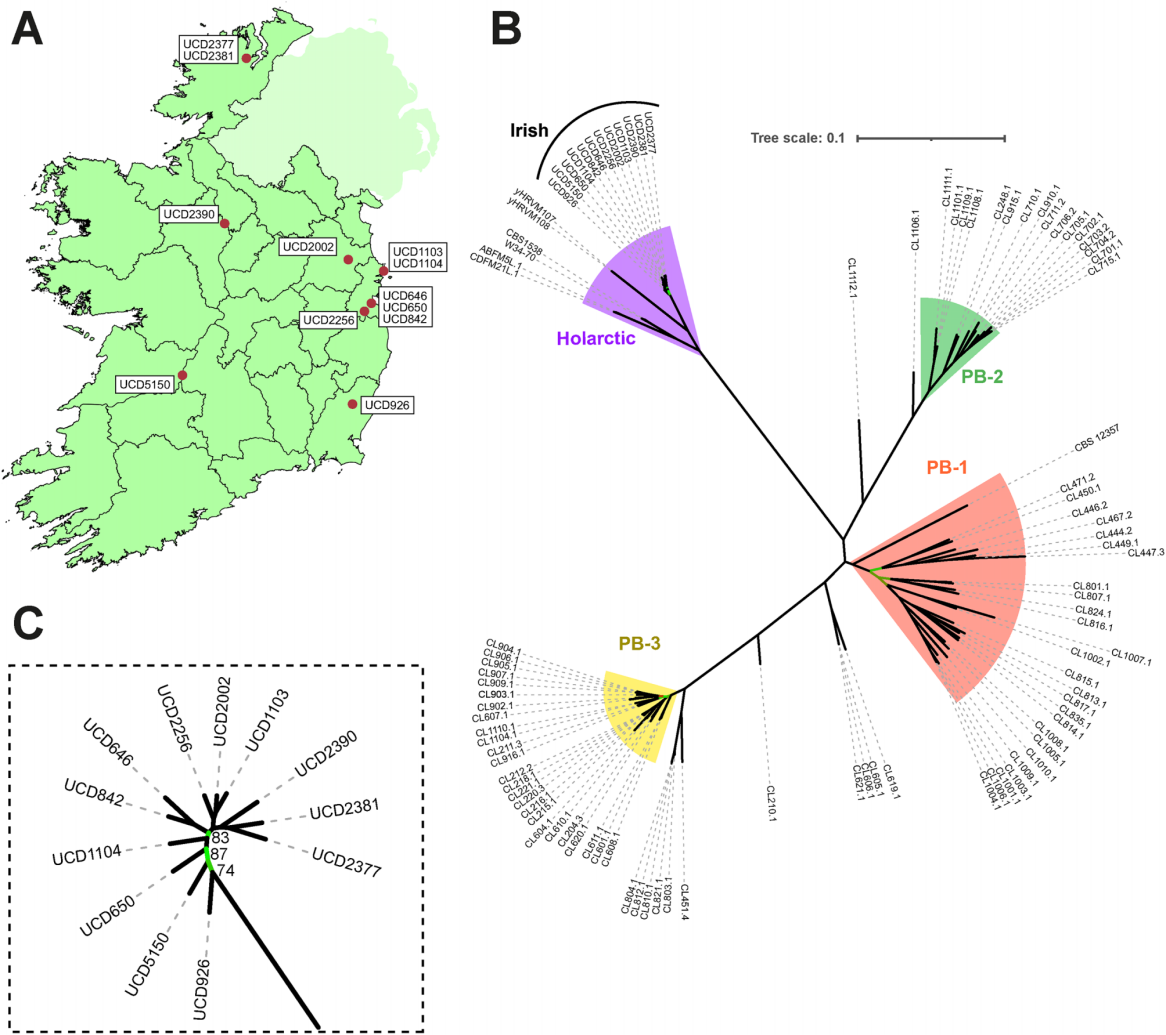
Isolation of *S. eubayanus* isolates from Ireland. (A) Map of Ireland marked with origin location of all Irish strains. (B) *Right:* SNP phylogeny of 12 Irish isolates, 85 other *S. eubayanus* strains, and the *S. eubayanus* component genomes of two *S. pastorianus* strains. The Holarctic clade and sub-clades of the Patagonia-B (PB) clade are marked with coloured arcs. The *S. eubayanus* type strain CBS 12357^T^ is labelled in bold. Bootstrap values under 90 are shown as coloured branches, ranging from 0 (red) to 90 (green). (C) Zoom-in to the Irish population. All bootstrap support values are 100 except for those marked.

In our previous study, *S. eubayanus* strains UCD646 and UCD650 were isolated from soil on the University College Dublin campus (Bergin et al. 2022). Subsequent analysis, mostly by undergraduate students (Ryan et al. 2025), identified ten new strains from soil near oak trees at seven sites across Ireland (Fig. 1A).

Whole-genome sequencing (short read) was used to characterize the genetic relationships between these strains and a larger *S. eubayanus* dataset (Supplementary Table 1). Phylogenomic analysis using single nucleotide polymorphism (SNP) alignment shows that the 12 Irish isolates cluster together in a specific subpopulation within the broader Holarctic clade, which also contains *S. pastorianus* (Fig. 1B). The Irish strains are very similar to each other with a mean pairwise SNP difference of 3020 sites (Supplementary Table 2), and we found that none of the new isolates could grow on maltose agar. In comparison, the mean number of SNPs between any Irish strain and the Patagonian type strain CBS 12357^T^ is 64 055.

**Figure 2:**
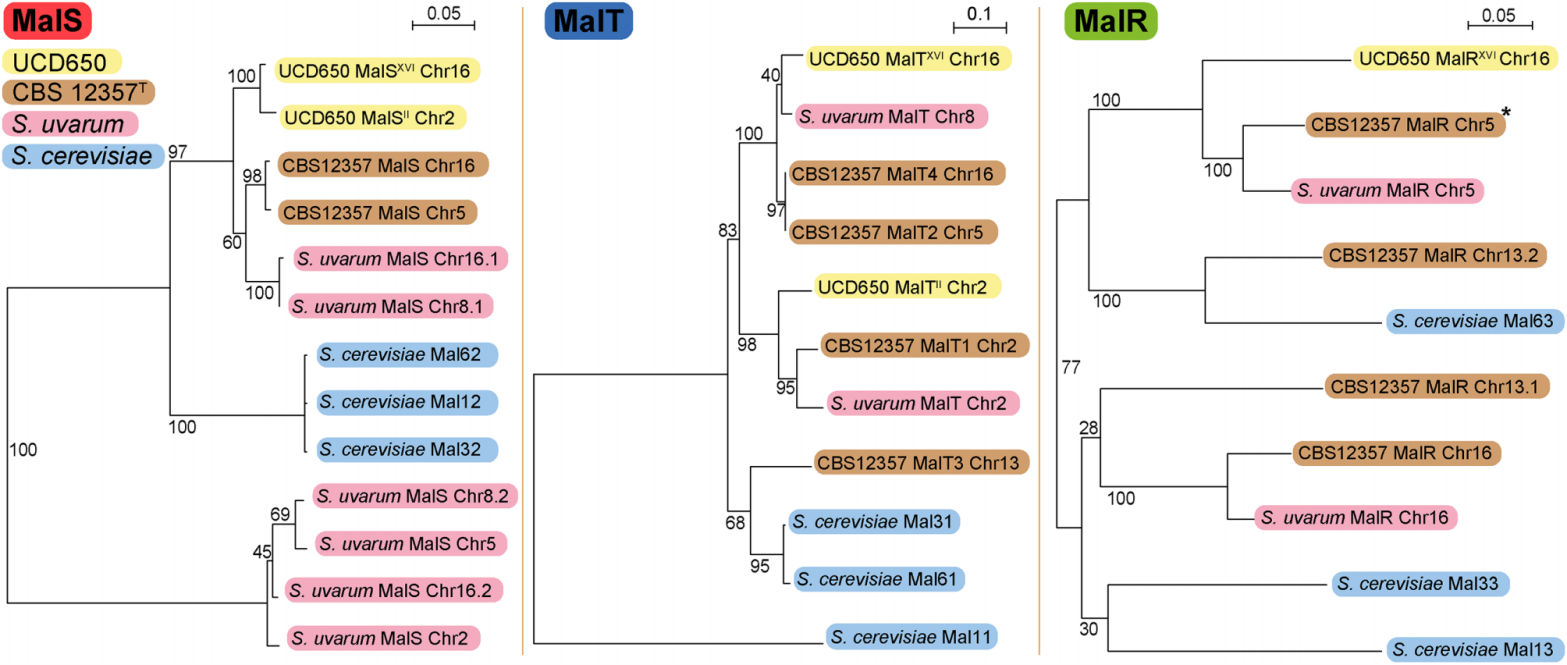
Phylogenetic comparison of Mal proteins. Protein trees of MalS (*left*), MalT (*centre*), and MalR (*right*) including all available sequences from UCD650, CBS 12357^T^, *S. uvarum* and the *MAL1, MAL3* and *MAL6* loci from *S. cerevisiae*. For CBS 12357, sequences were taken from the most up-to-date long read assembly of the strain (ASM332760v1). The MalR protein from CBS 12357^T^ marked with an asterisk has also been referred to as Mal33 (Quintrel et al. 2026). For *S. uvarum*, sequences were taken from the CBS 7001 long read assembly (GCA_947243805.1). For each protein family, the protein sequences were aligned using the Clustal Omega algorithm as implemented by Seaview (Gouy et al. 2010). The Gblocks program was used to identify informative sites for phylogenetic analysis, and trees were generated using PhyML within Seaview with 1000 bootstrap replicates for branch support and optimized estimation of invariable sites.

As previously reported (Bergin et al. 2022), the genome of UCD650 harbours a full complement of *MAL* genes organized in a *MAL* locus on chromosome XVI, as well as an extra copy of *MALS* and *MALT* on chromosome II. A frameshift error in the original UCD650 assembly, which suggested that *MALS^II^* (i.e. the *MALS* gene on chromosome II) was prematurely truncated, has been corrected. Phylogenetic analysis including the protein sequences of MalS, MalT, and MalR from UCD650, CBS 12357^T^, and other *Saccharomyces* species suggests that the three *MAL* genes have different evolutionary histories (Fig. 2).

For both *MALS* and *MALT*, the proteins encoded by the genes on chromosome XVI of UCD650 are closely related to the proteins from the same chromosome in CBS 12357^T^ (Fig. 2). In contrast, UCD650 MalR^XVI^ is more closely related to the copy of MalR from chromosome V of CBS 12357^T^ than it is to the one from chromosome XVI (Fig. 2). Even proteins encoded by the same locus have a different evolutionary history; in UCD650, MalS^II^ is highly similar to MalS^XVI^ but MalT^II^ and MalT^XVI^ are more distantly related (Fig. 2). Similarly, in CBS 12357^T^, the MalS and MalT proteins from chromosome V are near-identical to those from chromosome XVI while the MalR proteins from those chromosomes are more evolutionarily distant (Fig. 2). These data highlight the dynamic nature of the *MAL* genes, where duplications, deletions and gene conversions are common.

Interestingly, the MalS, MalR, and MalT1 proteins from CBS 12357^T^ are more closely related to the Mal proteins of the sister species *Saccharomyces uvarum* than they are to those of UCD650 (Fig. 2). This suggests that there may have been introgressions or gene sharing between Patagonian *S. eubayanus* strains and *S. uvarum* after the divergence of the Holarctic clade. Another possible explanation is that there could have been multiple paralogous *MAL* gene clusters in the most recent common ancestor of *S. eubayanus* and *S. uvarum*, of which some were retained in CBS 12357 and *S. uvarum* and others were retained in the Irish strains.

**Figure 3:**
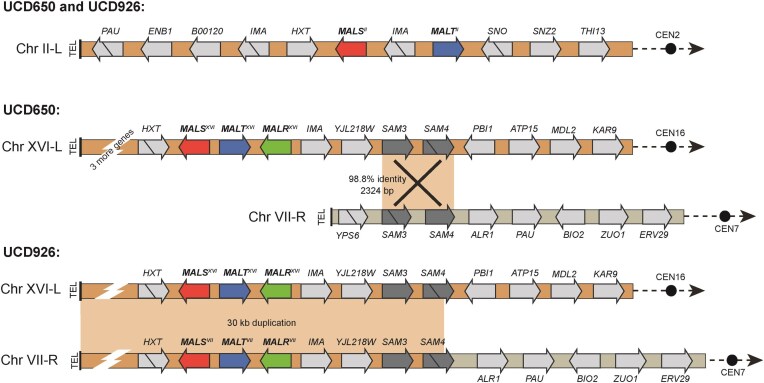
In wild *S. eubayanus* UCD926, the *MAL* locus of chromosome XVI is duplicated and replaces the right terminus of chromosome VII. Gene organization in subtelomeric regions at the left (L) or right (R) ends of chromosomes II, XVI, and VII is compared between the wild-type *S. eubayanus* isolates UCD926 and UCD650. *MAL* genes are coloured and named with superscripts indicating their chromosomal location. Orange background shading indicates regions of near-identical sequence on different chromosomes, and the X indicates the inferred site of the inter-chromosomal rearrangement that occurred in UCD926. Telomere (TEL) locations, and the direction toward the centromere (CEN), are indicated. Diagonal lines in gene symbols represent pseudogenes.

We also sequenced the genome of *S. eubayanus* UCD926 using Oxford Nanopore technology in conjunction with the short-read sequencing data to create an accurate, chromosome-length genome assembly and to characterize its *MAL* gene organization (Supplementary Table 3). The UCD926 genome is largely collinear with Irish isolates UCD646 and UCD650 whose genomes were described previously (Bergin et al. 2022). Like UCD650, there is an intact (three gene) *MAL* locus on chromosome XVI, copies of *MALS* and *MALT* on chromosome II (Fig. 3), as well as pseudogenized copies of *MALR* on chromosomes V and XIII. However, in addition a 30 kb region from the left end of chromosome XVI has become duplicated in UCD926 and replaces an 8 kb region at the right end of chromosome VII (Fig. 3). This rearrangement results in the gain of an entire copy of the *MAL^XVI^* locus (all three genes), and six neighbouring genes, at the right end of UCD926 chromosome VII, as well as the loss of a *YPS6* pseudogene (Fig. 3). The rearrangement was likely mediated by a 2.3 kb region of high (98.8%) sequence identity between the two chromosomes, spanning the genes *SAM3* and *SAM4* (Fig. 3). In addition, UCD926 also harbours a reciprocal translocation between chromosomes XI and XIII in comparison to other *S. eubayanus* genomes, with breakpoints occurring at common Ty4 elements (Supplementary Fig. 1). This rearrangement does not change gene copy numbers or cause disruption of any open reading frames.

### Evolution of maltose utilization in *S eubayanus* UCD650 and UCD926

Maltose and maltotriose are the main sugars in brewers’ wort (Otter and Taylor 1967, He et al. 2014). *Saccharomyces pastorianus* can metabolize both, and the *S. eubayanus* type strain CBS 12357^T^ and many wild isolates can utilize maltose (Mardones et al. 2020). As previously reported *S. eubayanus* UCD650 grows poorly in media containing maltose as the sole carbon source despite having a full component of *MAL* genes (Bergin et al. 2022). *Saccharomyces eubayanus* UCD926 also grows poorly in maltose medium despite having an extra copy of the *MAL* locus.

**Figure 4:**
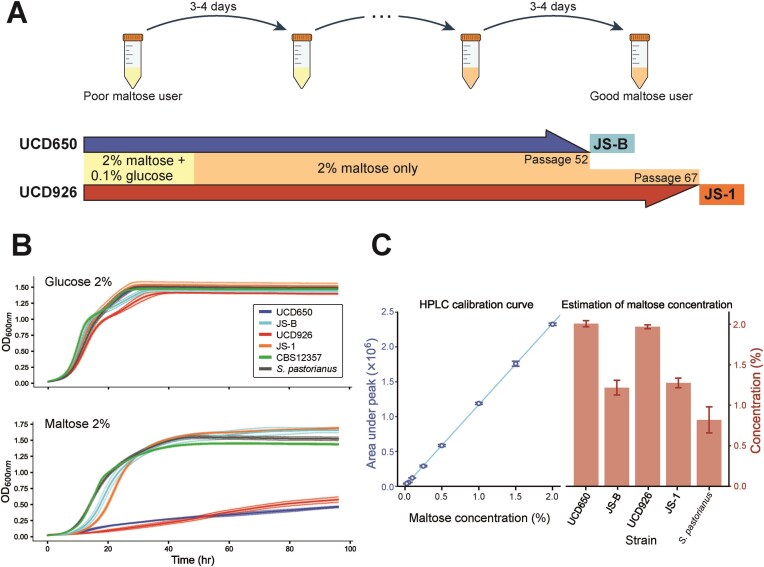
Adaptation of Irish *S. eubayanus* strains to growth in maltose in micro-evolution experiment. (A) Schematic of micro-evolution experiment (see Methods). (B) Growth rates of UCD650, UCD926, their respective evolved strains JS-B and JS-1, the type strain CBS 12357^T^, and *S. pastorianus* strain 7012 after 96 h growth in 2% glucose (top) and 2% maltose (bottom). Error bars show standard deviation of three biological replicates. (C) Consumption of maltose. *Left:* Increasing concentrations of maltose were used to define a HPLC calibration curve. Circles show the mean area under curve metric for seven concentrations of maltose, with whiskers showing the standard deviation of three technical replicates. *Right*: Mean estimated concentration of maltose remaining after growth of five strains with three replicates in SC medium + 2% maltose for 72 h. Whiskers show standard deviation of the three replicates for each strain.

Adaptive laboratory evolution was used to investigate the defect in maltose metabolism in the Irish *S. eubayanus* population, and to generate strains with brewing potential (Fig. 4A). Briefly, cultures of UCD650 and UCD926 were passaged every 3–4 days in YP medium containing 2% maltose + 0.1% glucose until growth stabilized, at which point they were passaged to a medium with 2% maltose only. At passage 52, one colony JS-B was randomly selected from the UCD650 lineage. Similarly, JS-1 was selected from the UCD926 lineage after passage 67. See Methods for full details.

Evolved strain JS-B grows better in maltose as the sole carbon source than its parental strain UCD650 as measured by the area under the curve (AUC) metric (*P*-value < 0.001, Welch’s t-test; Supplementary Table 4). Similarly, JS-1 grows better in maltose than its parent UCD926 (*P*-value < 0.001, Welch’s t-test; Supplementary Table 4). Both evolved strains have a shorter lag phase and reach higher optical densities, similar to *S. eubayanus* CBS 12357^T^ and *S. pastorianus* 7012. High-performance liquid chromatography (HPLC) showed that the concentration of maltose remaining in the culture medium following a 72 h incubation period was reduced to ∼1.25%, whereas it remained at 2% in the medium containing the parental isolates UCD650 and UCD926 (Fig. 4C). All *S. eubayanus* strains grew similarly on 2% glucose compared to the type strain, indicating that there is no general growth defect in either the wild-type or evolved strains (Fig. 4B).

### Evolved strains acquired both small- and large-scale genome changes

**Figure 5:**
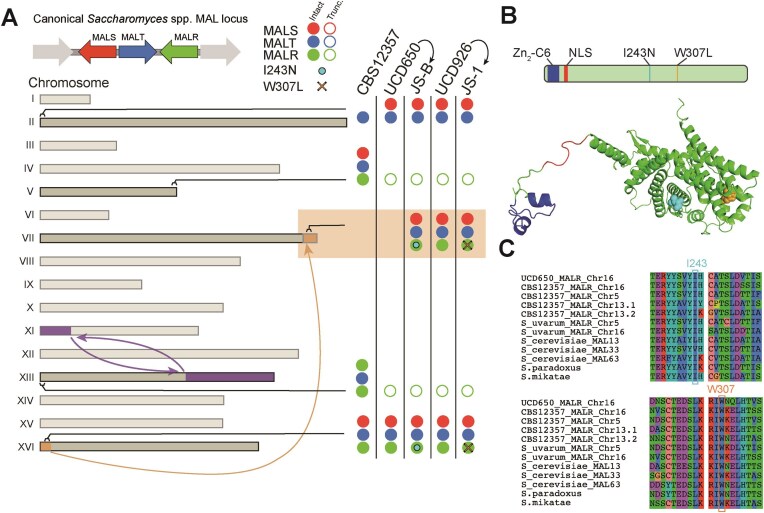
Evolved isolates acquired changes in *MALR* sequence and *MAL* locus copy number. (A) *Left*: Schematic of *S. eubayanus* chromosomes. Pointer arms extending from right of figure designate regions containing *MAL* genes at subtelomeric regions. The orange boxes and arrow show the duplication of a 30 kb region including a *MAL* locus, from the left end of chromosome XVI which has replaced the right end of chromosome VII in the evolved strain JS-B (but not its progenitor UCD650), and which is also present in wildtype UCD926 and its evolved strain JS-1. Purple boxes and arrows depict an additional reciprocal translocation between chromosome XI and XII in UCD926. *Right: MAL* gene content of each of the wild and evolved *S. eubayanus* isolates. Filled circles depict an intact gene at that locus, empty circles depict truncated genes, and no circle indicates an absence of the gene. Interior cyan circle and orange ‘X’ indicate *MALR* copies with the I243N and W307L mutations, respectively. (B) *Top:* Schematic of MalR protein. The blue box represents the Zn_2_-Cys_6_ fungal-type DNA binding domain, and the red box represents nuclear localization signal as determined by comparison to *S. cerevisiae* orthologs. Cyan and orange lines show the position of the two acquired mutations. *Bottom*: AlphaFold prediction of MalR structure, coloured as above. The I243 and W307 residues are shown as spheres for visual clarity. (C) Cropped snippets of alignment between the protein sequence of UCD650 MalR and orthologs in other *Saccharomyces* species. The blue and orange boxes highlight I243 and W307, respectively.

To determine what changes occurred in the genomes of the evolved strains during maltose adaptation, the genomes of JS-B and JS-1 were sequenced using both short- and long-read methods to generate high-quality genome assemblies (Supplementary Table 3). Strikingly, JS-B underwent a large genomic rearrangement during maltose adaptation, resulting in an additional copy of the entire *MAL* locus (all three genes) at the end of chromosome VII that is not present in its parent UCD650 (Fig. 5A). This rearrangement is very similar to the one present in the UCD926 parent (Fig. 3), which is maintained in the evolved JS-1 strain. The JS-B rearrangement breakpoints are indistinguishable from those in UCD926 and JS-1, indicating these independent rearrangement events likely used a common mechanism. Figure 5A summarises the organization of the *MAL* loci in the parental (UCD650, UCD926) and evolved (JS-B, JS-1) isolates in comparison to CBS 12357^T^.

The evolved strains acquired few nucleotide variants. The evolved strain JS-B acquired only six SNPs and one in-frame insertion relative to its parent UCD650, and the evolved strain JS-1 acquired only four SNPs relative to its parent UCD926 (Table 1). For comparison, there are more than 3000 SNP differences between JS-B and UCD926, which allows us to rule out the possibility that the duplication between chromosomes XVI and VII seen in JS-B is the result of contamination during the experiment; instead, it is the result of an independent rearrangement event that mimics the chromosomal structure naturally present in UCD926 and JS-1.

**Table 1:** List of small variants acquired by evolved strains.

	Position	Variant	Gene	*S. cerevisiae* Ortholog	Predicted effect
**JS-1**	ChrXVI:19 258	Hom.C→A	*U6460P00160*	*MALR/YPR196W*	Missense, W307L
	ChrXVI:681 343	Het.C→A	*U6460P03420*	*HOS1/YPR068C*	Upstream Variant
	ChrXV:684 472	Het.C→T	*U6460O03490*	*GND1/YHR183W*	Synonymous
	ChrV:541 781	Het.G→T	*U6460E02660*	*BMH1/YER177W*	Missense, G128C
**JS-B**	ChrXVI:19 450	Hom.A→T	*U6460P00160*	*MALR/YPR196W*	Missense, I243N
	ChrXVI:641 584	Het.G→T	*U6460P03180*	*TIP41/YPR040W*	Synonymous
	ChrXV:903	Het.G→C	*U6460O00110*	*MCH1/YKL221W*	Upstream Variant
	ChrXV:425 102	Het.A→T	*U6460O02170*	*FSH1/YHR049W*	Downstream Variant
	ChrXV:649 733	Het.C→A	*U6460O03320*	*PRP8/YHR165C*	Missense, R835L
	ChrX:119 298	Hom.C→T	*U6460J00640*	*VPS35/YJL154C*	Upstream Variant
	ChrIV:991 591	Het.T→TTAACCCATC	*U6460D05020*	*CSS1/YIL169C*	Conservative inframe insertion

Both of the evolved strains acquired mutations in *MALR*, and in both cases these mutations were the only homozygous variants identified in coding sequences (Table 1). JS-B acquired I243N amino acid substitutions in both MalR^XVI^ and MalR^VII^, changing the hydrophobic isoleucine residue to polar asparagine. JS-1 acquired W307L substitutions in both MalR^XVI^ and MalR^VII^, changing aromatic tryptophan to branch-chained leucine (Fig. 5B). *MALR^XVI^* and *MALR^VII^* are homozygous for the I243N mutation in JS-B and for W307L in JS-1. This was confirmed by mapping of the short read data (Supplementary Fig. 3A) and specific alignment of long-reads from either chromosome XVI or chromosome VII (Supplementary Fig. 3B). In the MalR protein, amino acid positions 243 and 307 are not close to the Zn_2_-Cys_6_ DNA binding domain and are therefore unlikely to directly affect the affinity of MalR for its target sequences (Fig. 5B). Instead, the recruitment of other regulatory factors that bind to the main body of the protein may be affected.

In MalR homologues from other *Saccharomyces* species, including the three *S. cerevisiae* MalR proteins, position 243 is consistently a branched-chain amino acid (isoleucine, leucine, valine). All paralogues of MalR in the *S. eubayanus* type strain CBS 12357^T^ have an isoleucine at this residue (Fig. 5C, Supplementary Fig. 2). Tryptophan at position 307 is conserved in all orthologues (Fig. 5C, Supplementary Fig. 2).

## Introduction of *MALR* mutations into UCD926 reproduces the maltose utilization phenotype

To determine whether the MalR substitutions I243N and W307L are sufficient to explain the increased ability of the evolved strains to utilize maltose, the two mutations were introduced individually into UCD650 and UCD926 via CRISPR-Cas9 editing, using a system developed for *S. cerevisiae* (Fleiss et al. 2019) that has also been used with *S. eubayanus* (Molinet et al. 2022). The W307L and I243N mutations were introduced into both alleles of *MALR^XVI^* and *MALR^VII^* in UCD650, generating strains UCD650_I243N_ and UCD650_W307L_ (Supplementary Fig. 4). Similarly, the W307L mutation was introduced into both alleles of *MALR^XVI^* and *MALR^VII^* of UCD926 to generate UCD926_W307L_. I243N was also introduced into UCD926, generating UCD926_I243N_. Sanger sequencing indicates that the I243N mutation is homozygous at one *MALR* gene and heterozygous at the other in UCD926_I243N_ (Supplementary Fig. 4).

**Figure 6:**
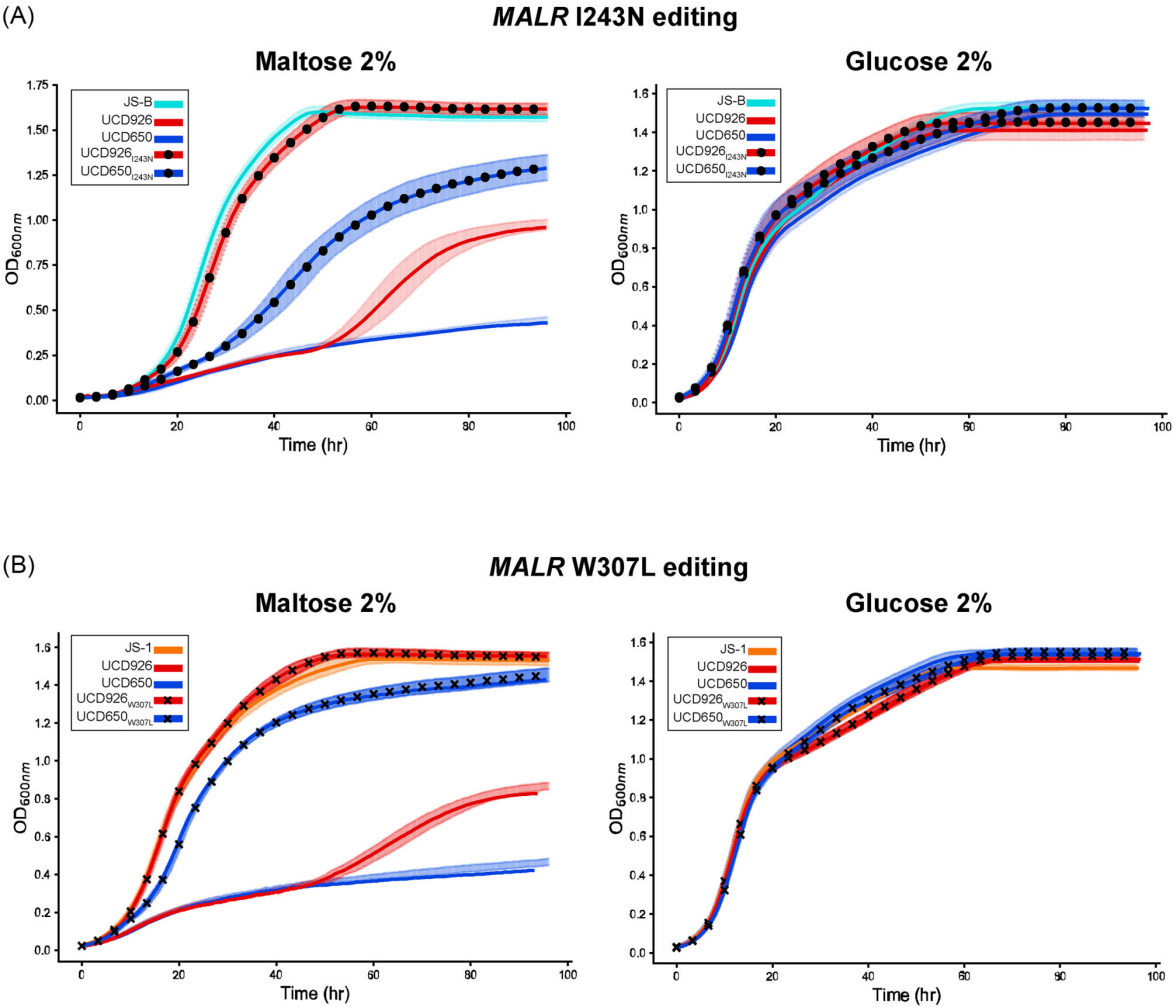
Introduction of *MALR* I243N and W307 L mutations recapitulate maltose phenotype. (A) I243N editing. *Left:* Growth of edited strains UCD650_I243N_ and UCD926_I243N_ compared to parental strains UCD650 and UCD926, and evolved strain JS-B (which contains I243N), in 2% maltose SC medium over 96 h as determined by absorbance values at 600 nm. *Right:* The same strains grown in 2% glucose medium over 96 h. (B) W307 L editing. *Left:* Growth of edited strains UCD650_W307 L_ and UCD926_W307 L_ compared to parental strains UCD650 and UCD926, and evolved strain JS-1 (which contains W307L), in 2% maltose SC medium over 96 h as determined by absorbance values at 600 nm. *Right:* The same strains grown in 2% glucose medium over 96 h.

Introduction of the I243N *MALR* mutation improved growth of both UCD650 and UCD926 on 2% maltose (Fig. 6A). Growth of UCD926_I243N_ is similar to the evolved strain JS-B (*P*-value = 0.148, Welch’s t-test; Supplementary Table 4), whereas UCD650_I243N_ has slower growth (*P*-value < 0.001, Welch’s t-test; Supplementary Table 4) and does not reach maximum levels after 96 h (Fig. 6A). For the W307L mutation, growth of UCD926_W307L_ is indistinguishable from the evolved strain JS-1 (*P*-value = 0.278, Welch’s t-test; Supplementary Table 4, Fig. 6B). UCD650_W307L_ grows less well, but still markedly better than parental UCD650 (*P*-value < 0.001, Welch’s t-test; Supplementary Table 4). UCD650_W307L_ grows faster than UCD650_I243N_ on maltose. It is therefore likely that the W307L mutation has a stronger effect on phenotype than I243N. It is also likely that the presence of the duplicated *MAL* locus in UCD926 but not UCD650 is necessary for maximum growth, irrespective of the MalR variant present.

UCD926 exhibited an increased growth rate after the 48-h time point (Fig. 6). This is also observed in Fig. 4B, though the difference is less pronounced in the latter. One possible explanation for this difference is that the room temperature varied in the two experiments; the average temperature was 20.4°C over 96 h in Fig. 6, and for Fig. 4B was an average of 24.1°C. All strains display similar growth in 2% glucose conditions (Fig. 6), showing that the acquired mutations are unlikely to affect basic glucose metabolism or impart a general growth defect under non-maltose conditions.

### Gain-Of-Function mutations in *MALR* affect expression of maltose-specific genes

Because *MALR* controls the expression of the maltase gene *MALS* and maltose transporter gene *MALT* (Charron et al. 1986), we hypothesized that I243N and W307L are Gain-Of-Function mutations that result in increased expression of *MALS* and *MALT*. Quantitative RT-PCR was used to test this hypothesis. All the *MAL* genes on chromosomes XVI and VII are identical and cannot be distinguished from each other. *MALS^II^* on chromosome II can also not be differentiated from *MALS* on chromosomes XVI and VII. However, expression of *MALT* on chromosome II (*MALT^II^*) can be differentiated from *MALT^XVI^* and *MALT^VII^*.

**Figure 7:**
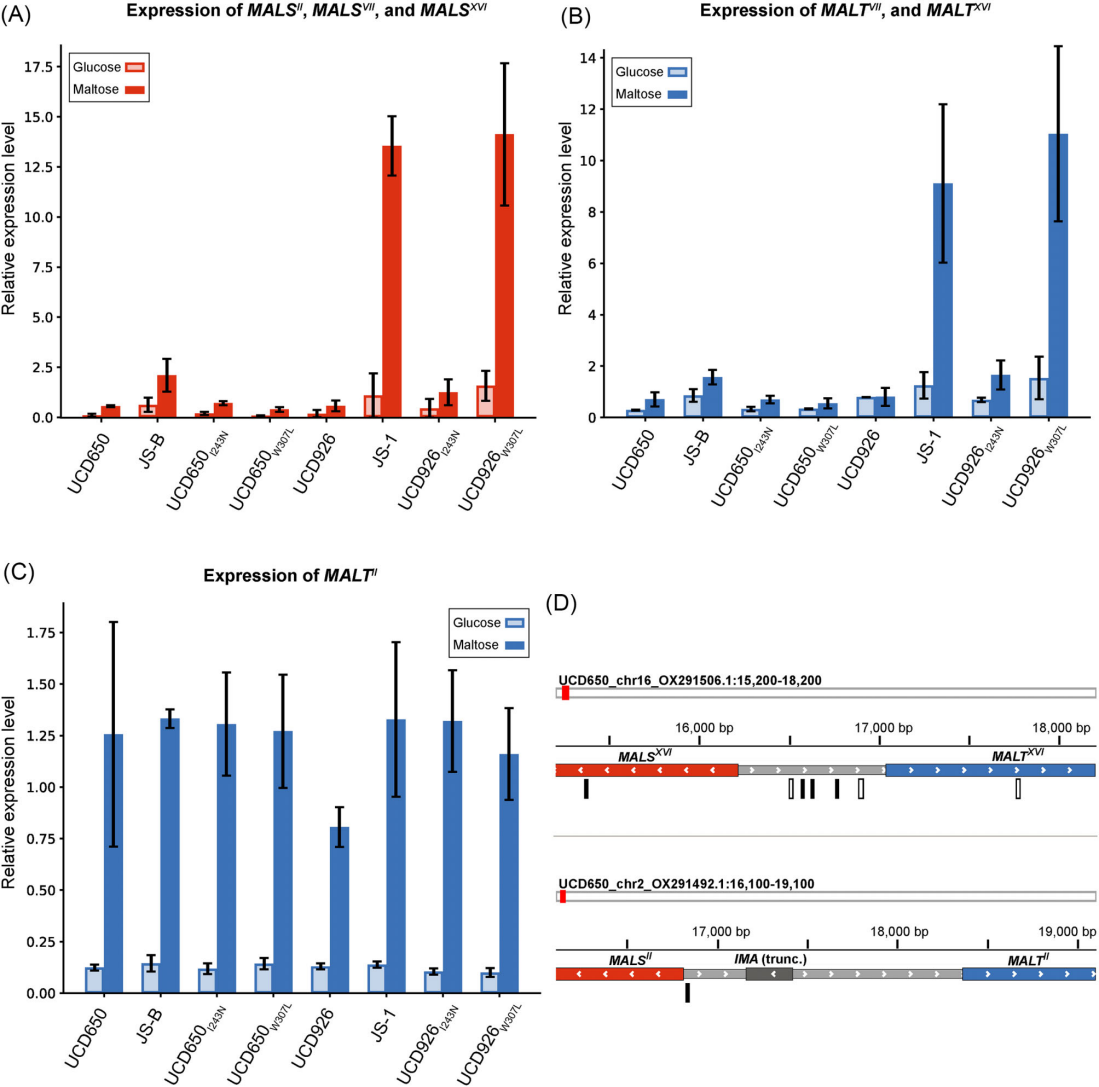
Introduction of the W307L mutation into the UCD926 background recapitulates the evolved phenotype. (A)–(C) Expression levels of various *MAL* genes across wild-type, evolved and CRISPR-edited strains after growth in either 2% glucose or 2% maltose media. Expression levels were normalized to the expression of the housekeeping gene *TAF10*. Coloured bars represent the mean of three biological replicates for each sample-target pair, and black whiskers show the standard deviation. (D) Edited IGV screenshot showing the relative positions of the 5′-CGSN_9_CGN-3′ motif to the *MAL* genes on chromosome XVI (*Top*) and chromosome II (*Bottom*). Black and white boxes represent the motif on the forward and reverse strands, respectively.

In wild-type strain UCD650, expression of the *MALS* and *MALT^XVI/VII^* genes show a marginal increase when the strain is grown in 2% maltose compared to 2% glucose (*MALS* p-value = 0.033, *MALT^XVI/VIII^ P*-value = 0.046, Welch’s t-test; Fig. 7A and B). There are no significant differences in expression of *MALS* and *MALT^XVI/VII^* in UCD926 between growth in maltose and growth in glucose (*MALS P*-value = 0.113, *MALT^XVI^ P*-value = 0.854, Welch’s t-test; Fig. 7A and B).

Both evolved strains JS-B and JS-1 have statistically significant higher levels of *MALS* expression during maltose growth when compared to their respective wild-type strains (Fig. 7A; Supplementary Table 5), and JS-1 also expresses *MALT^XVI/VIII^* to a higher level than UCD926 (Fig. 7B; Supplementary Table 5). The increase of expression is much higher in JS-1, which acquired the W307L mutation, than in JS-B which acquired the I243N mutation, despite both evolved strains having a *MAL* duplication (Fig. 7A and B). Introduction of either the I243N mutation or the W307L mutation into the UCD650 background, without the *MAL* locus duplication, did not result in significantly increased expression (Fig. 7A and B; Supplementary Table 5). In contrast, UCD926_I243N_ demonstrated a modest increase in expression of both *MALS* and *MALT^XVI/VII^* compared to wild-type UCD926, while introduction of the W307L mutation into UCD926 fully recapitulated the *MALS* and *MALT^XVI/VII^* expression seen in the evolved JS-1 strain (Fig. 7A and B; Supplementary Table 5).

The relative expression level of *MALT^II^* showed very little change across all strains, indicating that changes in *MALR* had no effect on this gene (Fig. 7C; Supplementary Table 5). In *S. cerevisiae* MalR activates *MALS* and *MALT* by binding to multiple copies of the motif 5′-CGSN_9_CGN-3′ present in the intergenic space between the two genes (Pougach et al. 2014). Binding to only a single copy of the motif is not sufficient for full gene activation (Pougach et al. 2014). In the Irish strains, there are five copies of this motif between *MALT^XVI^* and *MALS^XVI^* on chromosome XVI (Fig. 7D), which are maintained in the duplication on chromosome VII. At the chromosome II locus, there is only a single copy of the motif 18 bp upstream of the *MALT^II^* start codon, and no copies of the motif upstream of *MALS^II^* (Fig 7D). The 11-residue region of the *S. cerevisiae* MalR protein predicted to interact with the recognition site is fully conserved in UCD650 MalR^XVI^ (Pougach et al. 2014) (Supplementary Fig. 2). Neither of the evolved strains JS-1 or JS-B acquired mutations that disrupt this motif. Together these data suggest that the 5′-CGSN_9_CGN-3′ motif is likely functional in *S. eubayanus* and that the absence of multiple copies of the motif explains why expression of *MALT^II^* is not greatly affected by Gain-Of-Function mutations in *MALR*.

Altogether, the growth rate and expression data suggest that the combination of a *MAL* locus duplication, naturally occurring in UCD926 and acquired in JS-B, together with the acquisition of gain-of-function mutations in *MALR* account for the improved ability of the evolved strains to grow on maltose. The expression data also suggest that the phenotypic effect of the W307L mutations is greater than that of I243N, a result that is supported by growth rate analysis. Interestingly, despite having a noticeable effect on growth rate, introduction of the *MALR* mutations into UCD650 did not have a significant effect on expression of *MALS* and *MALT^XVI^*. It is possible that the observed phenotype change was mediated by expression changes in other genes not tested by RT-PCR, such as isomaltases.

## Discussion

To date, Ireland remains the only place in Europe in which *S. eubayanus* has been found. In our previous study (Bergin et al. 2022), we characterized two Irish isolates, UCD646 and UCD650, and showed that they were closely related to other members of the Holarctic clade and to the *S. eubayanus* component of the genome of the hybrid lager yeast *S. pastorianus*. Here we have reported ten more strains, from other locations around Ireland. Phylogenetically, all 12 Irish strains form a subclade within the Holarctic clade of *S. eubayanus*. The Irish isolates are all very similar to each other, unlike the *S. eubayanus* isolates in the deeply divergent Patagonian clades (Eizaguirre et al. 2018, Nespolo et al. 2020). This suggests that the Irish population resulted from a more recent expansion of the species onto the island, consistent with the Out-of-Patagonia migration hypothesis (Nespolo et al. 2020).

Both the original isolate UCD650 and the newly identified isolate UCD926 contain intact copies of maltase (*MALS*) and maltose transporter (*MALT*) genes, without frameshift or nonsense mutations. This suggests that their phenotype of poor growth on maltose is due to dysregulation of expression of these genes, even though the maltose regulatory gene (*MALR*) is also intact in these strains. Interestingly, strain UCD646 (collected at the same time as UCD650, at almost the same location) does not have any intact *MALR* genes; the copy in the chromosome XVI *MAL* locus acquired a frameshift mutation. Patagonian strain QC18, isolated from *Quercus rubor*, also acquired a frameshift truncation of a *MALR* gene (*MAL33^ChrV^*) leading to poor maltose metabolism (Quintrel et al. 2026). North American Holarctic strain yHRVM108 was isolated from *Pinus taeda* and also grows poorly on maltose (Baker and Hittinger 2019). These data suggest that the shift in environmental niche from association with maltose-rich *Nothofagus* species to the different sugar compositions of non*-Nothofagus* tree species allowed for a relaxation in selective pressure for maintenance of maltose metabolism genes. It has been shown that the strains from the Holarctic clade display higher numbers of structural variants than the Patagonian strains, which could be attributed to the Holarctic clade’s admixed origins (Villarreal et al. 2026). Structural variants that result in the loss of unnecessary genes, e.g. the loss of the chromosome V *MAL* locus, could be selected for in maltose-poor niches.

We found that laboratory selection of the Irish isolates for growth on maltose gave rise to two *de novo* point mutations in *MALR* with varying effects on the expression of *MALS* and *MALT*. It is possible that the few other mutations acquired during the course of the ALE also contribute to maltose metabolism phenotype, particularly in JS-B which acquired the I243N mutation that has a weaker effect. However, we believe that the convergent mutation of *MALR* in two distinct lineages, and the validation performed using CRISPR-Cas9 editing, is strong evidence for its role in the observed phenotypes. Previous investigations of the genomic causes of poor maltose growth in *S. eubayanus* strains have also identified *MALR* (*MALx3*) genes as a key determinant. One study found that expressing the intact *MALT* and *MALS* genes from a non-maltose utilizing *S. eubayanus* isolate from the Holarctic clade in a non-maltose utilizing *S. cerevisiae* strain enabled growth on maltose, indicating that both genes encode functional proteins (Brouwers et al. 2019). The researchers then showed that maltose growth could be restored to the non-maltose utilizing *S. eubayanus* strain by expressing a functional *MAL13* gene from *S. cerevisiae* (Brouwers et al. 2019). Quantitative trait locus mapping of offspring of two Patagonian strains (a maltose-capable strain CL467.1, and maltose-incapable strain QC18) identified the *MAL* loci on chromosomes XVI and V (Quintrel et al. 2026). Researchers were able to improve the maltose consumption phenotype in QC18 either by heterologous expression of the *MAL33^ChrV^* gene from CL467.1, or by overexpression of native QC18 *MAL33^ChrV^*, despite truncation of the gene (Quintrel et al. 2026). Interestingly, overexpression of QC18 *MAL33^ChrXVI^* had a weaker effect (Quintrel et al. 2026). A separate study showed that Irish and American strains unable to grow in maltose recovered growth when transformed with a copy of *MAL33^ChrV^* derived from CBS 12357^T^ (Villarreal et al. 2026). While the Irish strains do not have the chromosome V *MAL* locus, we observed that *MALR^XVI^* in Irish strains is more similar to CBS 12357^T^  *MAL33^ChrV^* than it is to *MAL33^ChrXVI^*. More work is needed to elucidate how the expression of different copies of *MALR* affects the expression of downstream maltase enzymes and maltose transporters.

Other studies have used an Adaptive Laboratory Evolution (ALE) approach to improve growth on maltose in maltose-deficient strains. Baker and Hittinger found that maltose-evolved isolates of a Holarctic (North American) strain yHRVM108 not only grew better on maltose, but also developed the ability to metabolize the trisaccharide maltotriose (Baker and Hittinger 2019). They attributed this to increased expression of the rare *AGT1* gene that encodes an ɑ-glucoside transporter in *S. pastorianus* and some members of the Holarctic *S. eubayanus* clade (Baker and Hittinger 2019). Follow-up work showed that this increased expression of *AGT1* was driven by a combination of cell-type switching by ploidy reduction to a haploid state, and the specific duplication of chromosome XV housing the *AGT1* gene (Crandall et al. 2023). Another study aimed at improving the maltose utilization of a low fermentation capacity Patagonian strain (CL815.1) found the evolved strain had acquired mutations in eight genes, none of which were *MAL* genes (Vega-Macaya et al. 2025). In this study we have described the first example of acquisition of Gain-Of-Function mutations in the *MALR* regulator and duplication of an entire *MAL* locus during ALE in *S. eubayanus* and showed that these genomic changes drive maltose growth via increased expression of the *MALS* and *MALT* genes.

Copy number variation is known to occur more frequently in subtelomeric regions in yeast species (Steenwyk and Rokas 2017) and these regions are often enriched for niche-specific genes such as the *MAL* genes (Snoek et al. 2013). By sequestering them to the subtelomeres, these loci can be more readily duplicated without the disadvantage that gene dosage effects can have on essential genes with more general functions. Here, we see a direct example of this increased evolvability of subtelomeric regions. JS-B duplicated a 30-kb region including a complete *MAL* locus, increasing its fitness in maltose-rich conditions without acquiring any general growth defect.

This study represents a first step toward the generation of a brewing-ready, maltose-consuming strain from wild Irish isolates of *S. eubayanus*. Evolved strains JS-1 and JS-B can metabolize 2% maltose at a rate similar to *S. pastorianus*, but higher concentrations of maltose similar to the brewing environment might require further adaptation. Additionally, the evolved strains (as well as other wild *S. eubayanus* strains) cannot metabolize maltotriose, which is a major component of the sugar composition of brewers’ wort. Multiple studies aiming to select for maltotriose consumption in *S. eubayanus* strains without *AGT1* have resulted in recombination between different copies of *MALT*, resulting in chimeric transporter genes that are capable of maltotriose intake (Baker and Hittinger 2019, Brouwers et al. 2019). Unlike the *S. eubayanus* strains used in these studies, Irish strains have only two *MALT* genes making similar adaptations more unlikely.

## Methods

### Isolation from soil

Soil samples were collected as previously described (Bergin et al. 2022). To identify species from single colonies, the ITS region of the rDNA locus was amplified by PCR using primers ITS1 and ITS4 (Supplementary Table 6). The PCR amplicon was sequenced with the Eurofins Genomics Mix2Seq platform.

### Short and long read sequencing

All strains were sequenced using Illumina short-read technologies. The genomes of UCD926, JS-1, and JS-B were also sequenced with Oxford Nanopore Technologies (ONT). For Illumina sequencing, genomic DNA was extracted using a phenol-chloroform-isoamyl alcohol method as described (Bergin et al. 2022). For strain UCD842, extracted DNA was prepared and sequenced by Novogene (UK) Company Ltd on a NovaSeq 6000 using the Illumina 300-cycle v1.5 kit on an S4 flow cell to a paired-end read length of 150 bp. For strains UCD926, UCD1102, UCD1004, and UCD2002, extracted DNA was used to prepare Illumina libraries using the Nextera DNA Flex Library Prep (Cat No. 20018704) and Illumina DNA/RNA UD Index Set A (Cat No. 20027213). Libraries were sequenced with NextSeq2000 in-house at University College Dublin. For the evolved strains JS-B and JS-1, libraries were prepared using the Illumina DNA prep (Cat No. 20 060 060/tagmentation), Illumina DNA/RNA UD Indexes Set A (Cat No. 20091646) and sequenced in-house using the Illumina NextSeq 2000 instrument using NextSeq 1000/2000 P1 Reagents (Cat No. 20050264). For strains UCD2256, UCD2377, UCD2381, UCD2390, and UCD5150, libraries were prepared with the TruSeq kit and sequenced by Novogene (UK) Company Ltd using a NovaSeq X Plus Series machine. UCD926 DNA was also sequenced with TruSeq Nano kit by Macrogen, Inc. using a NovaSeq X machine. All short-read sequencing generated paired-end reads of 2 × 150 bp.

For Nanopore sequencing, high molecular weight DNA was prepared using a MasterPure Yeast DNA Purification Kit (MPY80010; Biosearch Technologies) according to the manufacturer’s instructions. For UCD926, sequencing libraries were prepared from 1 µg of purified high molecular weight DNA using the Native Barcoding Sequencing Kit (SQK-NBD114-24; ONT). Libraries were sequenced using r10.4.1 chemistry flowcells (FLO-MIN114) on a MinION MK1C (ONT) device with MinKnow v. 22.10.7. Basecalling was performed using the fast configuration at default settings. Libraries were prepared in the same way for JS-1 and JS-B, but sequencing was performed on an MK1B (ONT) device using the super-accurate basecalling configuration in MiniKNOW v 24.02.8.

All sequencing data generated in the course of this study is available at accession PRJNA1454724.

### Genome assembly

UCD926 ONT MinION reads were filtered to remove reads with quality less than 7 and length less than 5 kb using NanoFilt (De Coster et al. 2018). For evolved strains JS-B and JS-1, cutoffs of quality < 7 and length < 1 kb were used instead. For all three strains, the filtered reads were assembled using Canu v2.2 (Koren et al. 2017) with parameters ‘genomesize = 13000000’. Assemblies were manually curated to remove small contigs. In order to reduce the number of errors introduced by error-prone MinION reads, the assemblies were polished with the short-read data using five rounds of NextPolish (Hu et al. 2020). The UCD926 assembly was manually edited to correct four frameshift errors in copies of *MALS* and *MALT* at chromosomes XVI and VII, verified by aligning long-reads using GraphMap v0.5.2 (Sović et al. 2016). Annotation of the assemblies was performed with YGAP (Proux-Wéra et al. 2012). BUSCO v6.1.0 was used to estimate assembly completeness using the saccharomycetes_odb12 lineage dataset (Supplementary Table 3) (Tegenfeldt et al. 2025), and QUAST v5.0.2 was used to generate assembly summary statistics (Gurevich et al. 2013).

### Variant analysis and phylogenetic tree construction

Short-read FASTQ files were trimmed for quality and to remove adaptors using Skewer v0.2.2 (Jiang et al. 2014) with parameters ‘-m pe -l 35 -q 30 -Q 30’. To identify reads from *S. pastorianus* that belong to the *S. eubayanus* sub-genome, trimmed reads were aligned against a concatenated FASTA file containing both the *S. eubayanus* UCD646 genome sequence (GCA_946408725.1) and the *S. cerevisiae* S288C genome (GCA_000146045.2) using the Burrows–Wheeler alignment tool. All reads that aligned to the S288C genome and all reads that had <90% identity to UCD646 were removed. GATK (v4.2.0.0) was used to mark duplicate reads and index subsequent BAM files (McKenna et al. 2010). Variants were called using the GATK HaplotypeCaller tool with mode ‘-ERC GVCF’, combined with GATK CombineGVCFs and genotyped with GATK GenotypeGVCFs. Variants were filtered using GATK VariantFiltration to remove calls with genotype quality <70, read depth <15, and to remove SNPs in clusters of five or more over a 20 bp span.

For phylogenetic analysis, biallelic SNPs were retained using GATK SelectVariants. A SNP alignment was generated for all 96 strains containing allele calls for every site in the genome that contained a called SNP in at least one strain using a custom script (http://github.com/CMOTsean/HetSiteRando). Heterozygous sites were randomly assigned to either reference or alternate allele on a site-by-site basis, ensuring all strains heterozygous at that site were assigned the same allele, and invariant sites were removed. A phylogeny was constructed using RAxML version 8.2.12 with the SNP alignment as input (Stamatakis 2014). RAxML was run using the ASC_GTRGAMMA model of nucleotide substitution with ‘–asc-corr lewis’ and with 1000 bootstrap replicates using ‘-# 54321 -p 54321’ as random seeds.

For identification of novel mutants in evolved strains, all variants filtered for quality were considered, including both SNPs and indels. All variants called in one of the evolved strains but not in their respective parent lineages were manually inspected and filtered. Variants which had a ‘no-call’ in either the evolved or parent strain were discarded.

### Adaptive evolution of UCD650 and UCD926 to maltose utilization

*Saccharomyces eubayanus* isolates UCD926 and UCD650 were streaked from stock onto YPD agar and incubated at room temperature for 4 days. Single colonies from each strain were inoculated into 10 ml of synthetic complete (SC) medium broth (0.17% Formedium Yeast Nitrogen Base without amino acids and ammonium sulphate, 0.079% Formedium Complete Supplement Mixture, 0.5% ammonium sulphate, 2% D-maltose monohydrate and 0.1% D-glucose), and incubated with shaking at 200 rpm at 25°C for 3 days.

Every 3–4 days, absorbance at 600 nm (OD_600_) of the cultures were measured and 1 ml of culture was passaged into 9 ml fresh SC 2% maltose/0.1% glucose medium. When absorbance values had plateaued, after 9 passages for UCD650 and 11 passages for UCD926, 1 ml of culture was passaged into SC 2% maltose broth without glucose. Cultures were further passaged in SC 2% maltose every 3–4 days until passage 52 for UCD650 and passage 67 for UCD926, at which point cultures were diluted and plated onto YPD agar. One colony was selected at random from each parental lineage (JS-1 for UCD926 and JS-B for UCD650) for further analysis and sequencing.

### Growth rates on glucose and maltose

Colonies were inoculated into 5 ml of YPD broth and incubated for 48 h at 200 rpm at room temperature. Three 1 ml volumes of each culture were centrifuged at 14 000 rpm for 1 min and pellets were washed three times in PBS (Phospho Buffered Saline) before being resuspended in SC 2% maltose or SC 2% glucose. Cultures were diluted to an OD_600_ of 0.1, and 200 μl was added to a 96 well plate. Absorbance was read every 10 min for 72 h using a SYNERGY/H1 microplate read (BioTek), version 30.04.17, double orbital continuous shaking at room temperature (ranged from ∼20°C to ∼24°C). The R package GrowthCurver was used to calculate exact area-under-the-curve (AUC) values for comparison (Sprouffske and Wagner 2016).

### High performance liquid chromatography

*S. eubayanus* strains UCD650 and UCD926, along with maltose evolved strains JS-1 and JS-B and *S. pastorianus* strain 7012 were streaked from stock to YPD and incubated at room temperature for 48 h. Three single colonies were inoculated into YPD broth and incubated at 20°C, 200 rpm for 48 h. Cultures were centrifuged at 4000 rpm for 5 min and pellets were washed twice in PBS. Pellets were resuspended in SC 2% maltose broth and diluted to an OD_600_ of 0.2 in fresh SC 2% maltose broth and incubated at 20°C with shaking at 200 rpm for 72 h. Cultures were centrifuged at 4000 rpm for 5 min and supernatants were filter sterilized. The HPLC was performed using an Agilent 1200 reverse phase HPLC with RID (set to 35°C) with column Phenomenex Luna 5 um NH2 100A (250 mm × 4.6 mm with 5 um particle size; part number 00g-4378-e0), which was kept at 30°C ± 0.8°C.

### CRISPR-Cas9 editing

CRISPR-Cas9 editing was carried out using plasmid pAEF5, which expresses *CAS9* from the *TEF1* promoter and the gRNA from the *SNR52* promoter (Fleiss et al. 2019). gRNAs were constructed by annealing and phosphorylating complimentary primers A and B (targeting I234N) or F and G (targeting W307L) (Supplementary Table 6), as described by Rajkumar and Morrissey (Rajkumar and Morrissey 2022). The resulting double-stranded gRNA inserts were diluted by 1:200 and cloned into 1 µl of pAEF5 (100 ng/µl) by Golden Gate Assembly at the SapI restriction site. Recombinant plasmids were transformed into *Escherichia coli* DH5a and screened by PCR using the M13 forward primer along with primers A or F (Supplementary Table 6). 120 bp repair templates were generated by primer extension using primers C and D (targeting I243N) or H and I (targeting W307L) (Supplementary Table 6) as described in Lombardi et al. (Lombardi et al. 2019).

*S. eubayanus* cells were grown overnight at 20°C with 200 rpm shaking in 5 ml of YPD broth, inoculated into fresh YPD in a total volume of 50 ml at an initial OD_600_ of 0.7, and incubated at 20°C with 200 rpm shaking for ∼4 h until an OD_600_ of 1.7 was reached. Cells were harvested by centrifugation at 4000 rpm (∼2000 x g) at 4°C (5 ml/transformation) and washed three times with 1 ml of ddH_2_O. The cell pellets were washed twice with 1 ml of 0.1 M lithium acetate. For each transformation, the washed cell pellet was resuspended in 240 µl of 50% (w/v) polyethylene glycol 3350, 36 μl of 1 M lithium acetate, and 20 μl of denatured salmon sperm (10 mg/ml). Total of 5 mg of the relevant pAEF5 plasmid and 25 μl of the corresponding repair template were added, vortexed briefly and incubated at 20°C for 45 min, followed by heat shock at 37°C for 30 min. Cells were pelleted, resuspended in 100 μl of YPD, and plated on YPD agar supplemented with 0.2 mg/ml hygromycin B. Plates were incubated at 20°C for 3–5 days.

Selected transformants were streaked onto fresh YPD agar. Edited strains were identified by colony PCR using the forward primer K with either primers E or J (Supplementary Table 6). The edits were confirmed by Sanger sequencing (using Eurofins Mix2Seq) of a larger amplicon (501 bp) generated using PCR with primers K and L (Supplementary Table 6).

### RT-PCR

RNA was extracted using the Qiagen RNeasy Mini Kit, treated with the Promega RQ1 RNase-Free DNase, and 40 ng of each was used in 10 μl reactions, performed in 384-well plates. The Promega 1-Step RT-qPCR Kit was used, and the plates were assembled using an Opentrons OT-2 Robot. The assays were run in the Real-time PCR system QuantStudio 5 with the following protocol: 37°C for 15 min; 95°C for 10 min; 40x (95°C for 30 s, 60°C for 30 s, 72°C for 30 s), and melting curve: 95°C for 10 s; 65°C for 60 s; 97°C for 1 s.

Cq values were used to calculate relative expression levels. The median Cq of two technical replicates were used for each target gene compared to the housekeeping gene *TAF10*. Relative expression was found using the formula 2^−(Cq(Target_gene)-Cq(TAF10)^. The mean and standard deviation of the three biological replicates were plotted. ANOVA and post-hoc Tukey’s Honestly Significant Difference (HSD) test were used to identify statistical significance in the differences between the mean ΔCq values of strains grown on maltose for each of the three genes tested.

## Supplementary Material

foag036_Supplemental_Files
