# Supplementary material for: Adaptive laboratory evolution of maltose utilization in wild Irish isolates of Saccharomyces eubayanus
Source: FEMS Yeast Res. 2026 Aug 8;26:foag036. doi: 10.1093/femsyr/foag036 (PMC13479922; doi:10.1093/femsyr/foag036)
Supplement: Supplementary file 1 [file foag036_supplemental_files.zip › Bergin_Eubayanus_Supplementary_Figures.pdf]

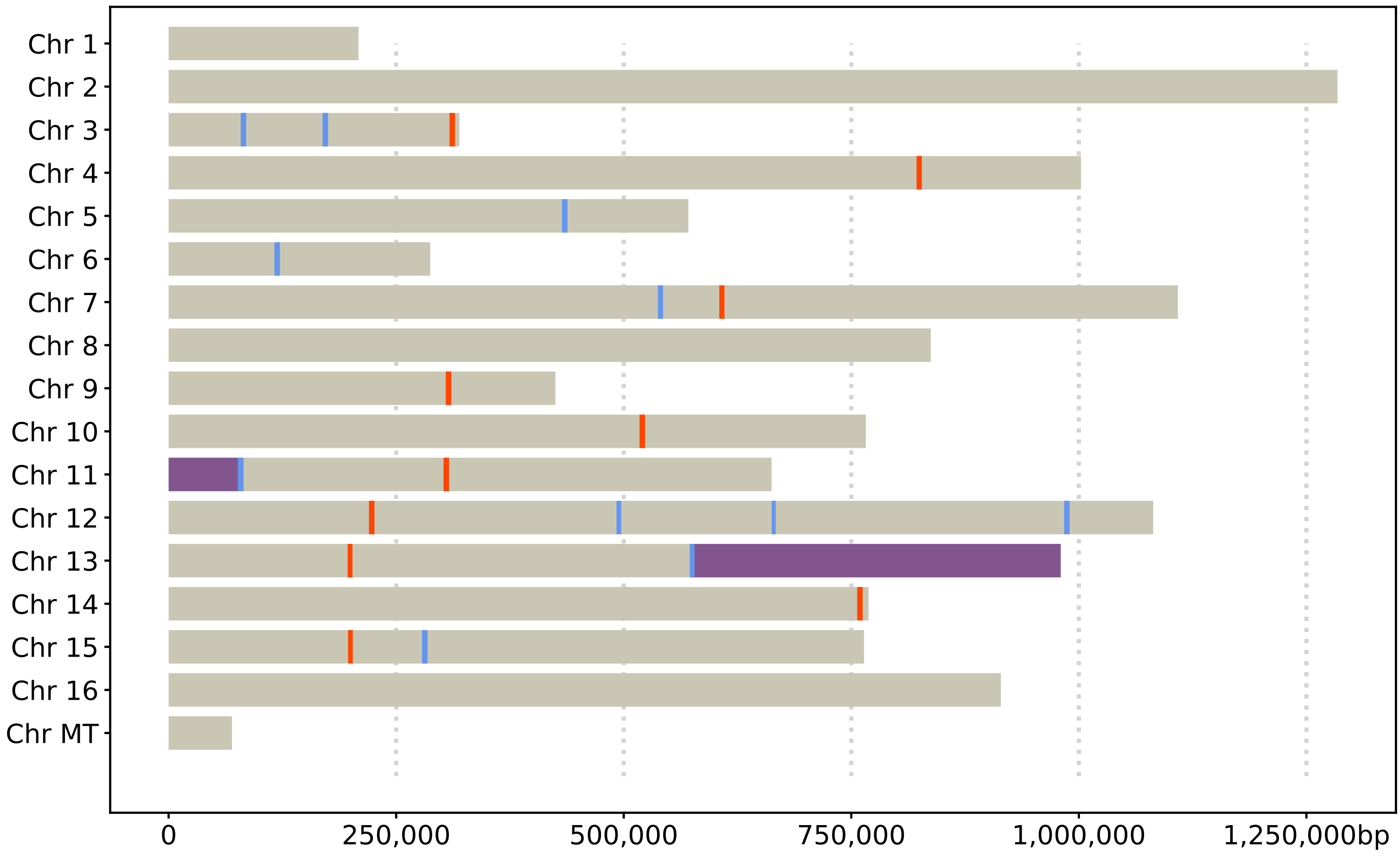

Supplementary Figure 1: Location of Ty4 elements in the UCD650 genome identified by BLAST. Each chromosome is represented by a gray bar. Purple bars highlight the regions on chromosomes XI and XIII that underwent reciprocal translocation to form recombinant chromosomes in UCD926. Blue lines represent Ty4 elements on the forward strand and red lines show elements on the reverse.

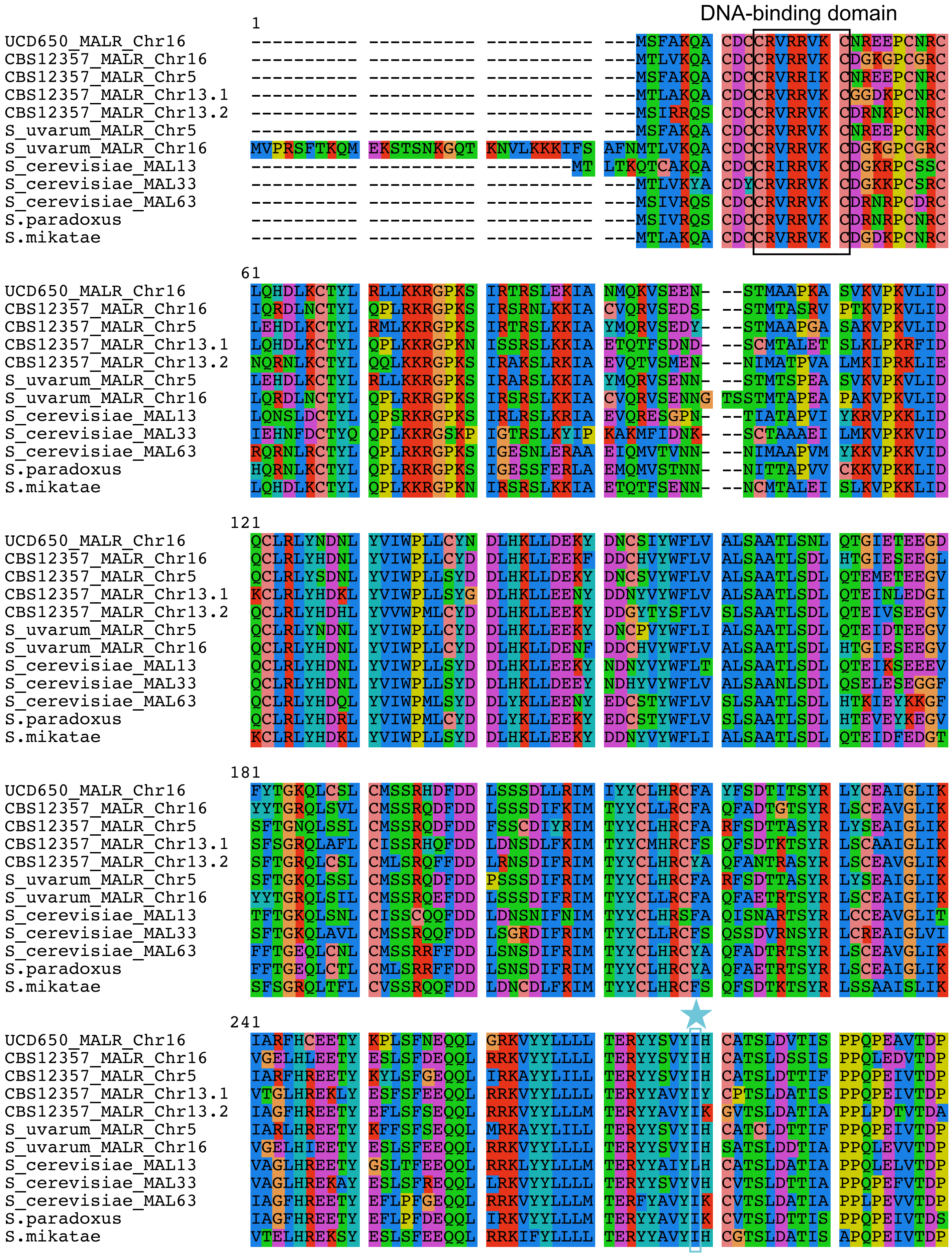

DNA-binding domain
1
UCD650_MALR_Chr16 ---------- ---------- ---------- ---MSFAKQA CDCCRVRRVK CNREEPCNRC
CBS12357_MALR_Chr16 ---------- ---------- ---------- ---MTLVKQA CDCCRVRRVK CDGKGPCGRC
CBS12357_MALR_Chr5 ---------- ---------- ---------- ---MSFAKQA CDCCRVRRIK CNREEPCNRC
CBS12357_MALR_Chr13.1 ---------- ---------- ---------- ---MTLAKQA CDCCRVRRVK CGGDKPCNRC
CBS12357_MALR_Chr13.2 ---------- ---------- ---------- ---MSIRRQS CDCCRVRRVK CDRNKPCNRC
S_uvarum_MALR_Chr5 ---------- ---------- ---------- ---MSFAKQA CDCCRVRRVK CNREEPCNRC
S_uvarum_MALR_Chr16 MVPRSFTKQM EKSTSNKGQT KNVLKKKIFS AFNMTLVKQA CDCCRVRRVK CDGKGPCGRC
S_cerevisiae_MAL13 ---------- ---------- --------MT LTKQTCAKQA CDCCRIRRVK CDGKRPCSSC
S_cerevisiae_MAL33 ---------- ---------- ---------- ---MTLVKYA CDYCRVRRVK CDGKKPCSRC
S_cerevisiae_MAL63 ---------- ---------- ---------- ---MSIVRQS CDCCRVRRVK CDRNRPCDRC
S.paradoxus ---------- ---------- ---------- ---MSIVRQS CDCCRVRRVK CDRNRPCNRC
S.mikatae ---------- ---------- ---------- ---MTLAKQA CDCCRVRRVK CDGDKPCNRC
61
UCD650_MALR_Chr16 LQHDLKCTYL RLLKKRGPKS IRTRSLEKIA NMQKVSEEN- --STMAAPKA SVKVPKVLID
CBS12357_MALR_Chr16 IQRDLNCTYL QPLRKRGPKS IRSRNLKKIA CVQRVSEDS- --STMTASRV PTKVPKVLID
CBS12357_MALR_Chr5 LEHDLKCTYL RMLKKRGPKS IRTRSLKKIA YMQRVSEDY- --STMAAPGA SAKVPKVLID
CBS12357_MALR_Chr13.1 LQHDLKCTYL QPLKKRGPKN ISSRSLKKIA ETQTFSDND- --SCMTALET SLKLPKRFID
CBS12357_MALR_Chr13.2 NQRNLKCTYL QQLKKRGPKS IRAKSLRKIA EVQTVSMEN- --NIMATPVA LMKIPRKLIE
S_uvarum_MALR_Chr5 LEHDLKCTYL RLLKKRGPKS IRARSLKKIA YMQRVSENN- --STMTSPEA SVKVPKVLID
S_uvarum_MALR_Chr16 LQRDLNCTYL QPLRKRGPKS IRSRSLKKIA CVQRVSENNG TSSTMTAPEA PAKVPKVLID
S_cerevisiae_MAL13 LQNSLDCTYL QPSRKRGPKS IRLRSLKRIA EVQRESGPN- --TIATAPVI YKRVPKKLID
S_cerevisiae_MAL33 IEHNFDCTYQ QPLKKRGSKP IGTRSLKYIP KAKMFIDNK- --SCTAAAEI LMKVPKKVID
S_cerevisiae_MAL63 RQRNLRCTYL QPLRKRGPKS IGESNLERAA EIQMVTVNN- --NIMAAPVM YKKVPKKVID
S.paradoxus HQRNLKCTYL QPLRKRGPKS IGESSFERLA EMQMVSTNN- --NITTAPVV CKKVPKKLID
S.mikatae LQHDLKCTYL QPLKKRGPKN IRSRSLKKIA ETQTFSENN- --NCMTALEI SLKVPKKLID
121
UCD650_MALR_Chr16 QCLRLYNDNL YVIWPLLCYN DLHKLLDEKY DNCSIYWFLV ALSAATLSNL QTGIETEEGD
CBS12357_MALR_Chr16 QCLRLYHDNL YVIWPLLCYD DLHKLLEEKF DDCHVYWFLV ALSAATLSDL HTGIESEEGL
CBS12357_MALR_Chr5 QCLRLYSDNL YVIWPLLSYD DLHKLLDEKY DNCSVYWFLV ALSAATLSDL QTEMETEEGV
CBS12357_MALR_Chr13.1 KCLRLYHDKL YVIWPLLSYG DLHKLLEENY DDNYVYWFLV ALSAATLSDL QTEINLEDGI
CBS12357_MALR_Chr13.2 QCLRLYHDHL YVVWPMLCYD DLHKLLEEKY DDGYTYSFLV SLSAATLSDL QTEIVSEEGV
S_uvarum_MALR_Chr5 QCLRLYNDNL YVIWPLLCYD DLHKLLEEKY DNCPVYWFLI ALSAATLSDL QTEIDTEEGV
S_uvarum_MALR_Chr16 QCLRLYHDNL YVIWPLLCYD DLHKLLDENF DDCHVYWFLV ALSAATLSDL HTGIESEEGV
S_cerevisiae_MAL13 QCLRLYHDNL YVIWPLLSYD DLHKLLEEKY NDNYVYWFLT ALSAATLSDL QTEIKSEEEV
S_cerevisiae_MAL33 QCLRLYHDNL YVIWPLLSYD DLHKLLDEEY NDHYVYWFLV ALSAANLSDL QSELESEGGF
S_cerevisiae_MAL63 QCLRLYHDQL YVIWPMLSYD DLYKLLEENY EDCSTYWFLV SLSAATLSDL HTKIEYKKGF
S.paradoxus QCLRLYHDRL YVIWPMLCYD DLYKLLEEKY EDCSTYWFLV SLSAATLSDL HTEVEYKEGV
S.mikatae KCLRLYHDKL YVIWPLLSYD DLHKLLEEKY DDNYVYWFLI ALSAATLSDL QTEIDFEDGT
181
UCD650_MALR_Chr16 FYTGKQLCSL CMSSRHDFDD LSSSDLLRIM IYYCLHRCFA YFSDTITSYR LYCEAIGLIK
CBS12357_MALR_Chr16 YYTGRQLSVL CMSSRQDFDD LSSSDIFKIM TYYCLLRCFA QFADTGTSYR LSCEAVGLIK
CBS12357_MALR_Chr5 SFTGNQLSSL CMSSRQDFDD FSSCDIYRIM TYYCLHRCFA RFSDTTASYR LYSEAIGLIK
CBS12357_MALR_Chr13.1 SFSGRQLAFL CISSRHQFDD LDNSDLFKIM TYYCMHRCFS QFSDTKTSYR LSCAAIGLIK
CBS12357_MALR_Chr13.2 SFTGRQLCSL CMLSRQFFDD LRNSDIFRIM TYYCLHRCYA QFANTRASYR LSCEAVGLIK
S_uvarum_MALR_Chr5 SFTGKQLSSL CMSSRQDFDD PSSSDIFRIM TYYCLHRCFA RFSDTTASYR LYSEAIGLIK
S_uvarum_MALR_Chr16 YYTGRQLSIL CMSSRQEFDD LSSSDIFRIM TYYCLLRCFA QFAETRTSYR LSCEAIGLIK
S_cerevisiae_MAL13 TFTGKQLSNL CISSCQQFDD LDNSNIFNIM TYYCLHRSFA QISNARTSYR LCCEAVGLIT
S_cerevisiae_MAL33 SFTGKQLAVL CMSSRQQFDD LSGRDIFRIM TYYCLLRCFS QSSDVRNSYR LCREAIGLVI
S_cerevisiae_MAL63 FFTGEQLCNL CMSSRRFFDD LSNSDIFRIM TYYCLHRCYA QFADTRTSYR LSCEAVGLIK
S.paradoxus FFTGEQLCTL CMLSRRFFDD LSNSDIFRIM TYYCLHRCYA QFAETRTSYR LSCEAIGLIK
S.mikatae SFSGRQLTFL CVSSRQQFDD LDNCDLFKIM TYYCLHRCFS QFSDTKTSYR LSSAAISLIK
241
UCD650_MALR_Chr16 IARFHCEETY KPLSFNEQQL GRKVYYLLLL TERYYSVYIH CATSLDVTIS PPQPEAVTDP
CBS12357_MALR_Chr16 VGELHLEETY ESLSFDEQQL RRKVYYLLLL TERYYSVYIH CATSLDSSIS PPQLEDVTDP
CBS12357_MALR_Chr5 IARFHREETY KYLSFGEQQL IRKAYYLILL TERYYSVYIH CATSLDTTIF PPQPEIVTDP
CBS12357_MALR_Chr13.1 VTGLHREKLY ESFSFEEQQL RRKVYYLLLL TERYYAVYIY CPTSLDATIS PPQPEIVTDP
CBS12357_MALR_Chr13.2 IAGFHREETY EFLSFSEQQL RRKVYYLLLM TERYYAVYIK GVTSLDATIA PPLPDTVTDA
S_uvarum_MALR_Chr5 IARLHREETY KFFSFSEQQL MRKAYYLILL TERYYSVYIH CATCLDTTIF PPQPEIVTDP
S_uvarum_MALR_Chr16 VGELHIEETY ESLSFEEQQL RRKVYYLLLL TERYYSVYIH SATSLDDTIA PPQLEVVTDP
S_cerevisiae_MAL13 VAGLHREETY GSLTFEEQQL RRKLYYLLLM TERYYAIYLH CATSLDATIA PPQLELVTDP
S_cerevisiae_MAL33 VAGLHREKAY ESLSFREQQL LRKVYYLLLL TERYYSVYVH CVTSLDTTIA PPQPEFVTDP
S_cerevisiae_MAL63 IAGFHREETY EFLPFGEQQL RRKVYYLLLM TERFYAVYIK CVTSLDATIA PPLPEVVTDP
S.paradoxus IAGFHREETY EFLPFDEQQL IRKVYYLLLM TERYYAVYIK CVTSLDTTIS PPQPEIVTDS
S.mikatae VTELHREKSY ESLSFEEQQL RRKIFYLLLL TERYYAVYIH CGTSLDATIS APQPEIVTDP

```
                       301
UCD650_MALR_Chr16      RLSLDSFLEV IGVFTVPEKC FFDALATGPA DNSCTEDSLK RIWNQLHTVS PDVEPWSYGY
CBS12357_MALR_Chr16    RLSLDSFLEM IRVFTVPGKY FFDALASNSE NVSCTEDSLK KIWKELHTSS LEIEPWSYGY
CBS12357_MALR_Chr5     RLSLDSFLEV IRVFTVPEKC FYDSLATDFA NDSCTEDSLK RIWKELHTVS PEVEPWSYGY
CBS12357_MALR_Chr13.1  RLSLDSFLEM IRVFTVPGKC FFDALATDSS DASCTEDSLK RIWKELHTTS LEIEPWSYGY
CBS12357_MALR_Chr13.2  RLSLDSFLEV IRVFTVPGKC FYDALATNCA NNSCTEDSLK RIWNELHTAS LNIEPWSYGY
S_uvarum_MALR_Chr5     RLSLDSFLEV IRVFTVPEKC FYDALATDFA NDSCTEDSLK RIWKDLYTIS PEVEPWSYGY
S_uvarum_MALR_Chr16    RLSLDSFLEM IRVFTIPGKC FFDALATDSE NVSCTEDSLK KIWKELHTSS LEIEPWSYGY
S_cerevisiae_MAL13     QLSMDSFLEM IRVFTVPGKC FFDALAADST DASCTEESLK KIWNELHTTS SEIEPWSNGY
S_cerevisiae_MAL33     RLSLDSFFEM IRVFTVPGKC FFDALATEST SGSCTEDSLK KIWKELHTAS LEIEPWSYGY
S_cerevisiae_MAL63     RLSLESFLEV IRVFTIPGKC FYDALATNCV DDSYTEDSLK RIWNELHTTS LDIEPYSYGY
S.paradoxus            RLSLDSFLEV IRVFTVPGKY FYDALATNSV NDSYTEDSLK RIWNELHTTS LAIEPCSYGY
S.mikatae              RLSLDSFLEM IRVFTVPGKC FFDALATDSS NSSCTEDSLK RIWKELHTAS LEIEPWSYGY

                       361
UCD650_MALR_Chr16      VDFSFSRHWI RTLAWKLMFL AKGKQTKFLL NTHNAHIPVQ IAKDMLEDTF LTPKNFYDVH
CBS12357_MALR_Chr16    VDISFSRHWI RALAWRLVFR MKG--ANFFS NSNNAHIPVE IARDMLDDIF LSPNNLYEVH
CBS12357_MALR_Chr5     IDFLFSRHWI RTLAWKLMFQ VKDKRTKFLS NTDNAHIPVE IARDMLGDTF LTPKNFYDVH
CBS12357_MALR_Chr13.1  VDISFSRHWI RILAWKLVFR TKS--INSLS ASSNAQIPVE IARDMLDDVF LTPANLYGVH
CBS12357_MALR_Chr13.2  VDSLFSRHWV RTLAWKLVLH MKGMRINFLS NTNNTHIPVE IARDMLGDTF LTAKNIYDVH
S_uvarum_MALR_Chr5     VDFLFSRHWI RTLAWKLMFQ VKDKQTKFLS NADNAHIPVK IARDMLGDTF LTPKNSYDVH
S_uvarum_MALR_Chr16    VDISFSRHWI RALAWRLVFR MKG--ANFFS NSNNAHIPVE IARDMLDDIF LSPNNLYEVH
S_cerevisiae_MAL13     IDISFSRHWI RILAWKLAYQ MRG--SNFSL NANNGQIPIE IARDMLIDTY LTPENLYDVH
S_cerevisiae_MAL33     VDISFSRHWI RALAWKLVFQ MNG--TKFFS NANNAHILVE IAKDMLDDIF LTPNNLYDVH
S_cerevisiae_MAL63     IDYLFSRHWV RTLAWKLVLN KKDMRMNFFS NTNVTHIPVE IAKDMLQDTL LTPIDLYDVH
S.paradoxus            IDYHFSRHWV RTLAWKLVLH KKGMRLNLFS NTIVTHIPVE IAKDMLRDTL LTPIDLYDVH
S.mikatae              VDISFSRHWI RVLAWRLVFQ TKS--INFLS TSNNAQIPVE IARDMLDDVF LTPANLYGVH

                       421
UCD650_MALR_Chr16      GPGIPIKVLE IANALVDVVN HYDQNMELEA WNVLRDVVKF VFSLKYCDPN MIQKFTTKCQ
CBS12357_MALR_Chr16    GPGISTKALE IANALVDVVK QYDQDIESEA WSTLCDVVKF VFSLKQCDNK MIQRFSTKCQ
CBS12357_MALR_Chr5     GPGIPMKTLE IANALVDVIG HYDRNMKLEA WNVLSDVAKF VFSLNHCDNI MFQRFSTKCQ
CBS12357_MALR_Chr13.1  GPGIPAKALE VANALVDVVN QYDQNTESEA WKVLCEISKF VFSLKHYDGK LVESFVTKCQ
CBS12357_MALR_Chr13.2  GPGIPMKALE VANALVDVVS KYDRNMKLEA WNVLYDVSKF VFSLKHCNNE MVQKFSSKCQ
S_uvarum_MALR_Chr5     GPGIPMKTLE IANALVDVIN HYDQNMKLEA WNVLCDVVKF VFSLKHCDNK MFQRFSVKCQ
S_uvarum_MALR_Chr16    GPGISTKALE IANALVDVVK QYDQDVESEA WSALCDVVKF VFSLKHRDNK MIQRFSTKCQ
S_cerevisiae_MAL13     GPGVPVKTLE IATALVDIVG QYDHNMKLEA WNVLHDVCKF AFSLNHYNND MLKRFSTKCQ
S_cerevisiae_MAL33     GPGIPMKSLE VANALVDIVN KYDHNMKLEA WNILCDVSKF VFSLKHCNHK MFQRFSTKCQ
S_cerevisiae_MAL63     GPGVPMKALE IANALVDVVS KYDHNMKLEA WNILCDVSKF VFSLKHCNHK MFQRFSTKCQ
S.paradoxus            GPGIPMKALE IANALIDVVS KYDHNMKLEA WNVLCDVSKF VFSLKHCNHK MFQRFSTKCQ
S.mikatae              GPGIPAKALE VANALVDVVN QYGQNTESEA WKVLCEISKF VFSLKHYDGK LVESFVTKCQ

                       481
UCD650_MALR_Chr16      SALITLPITR LLQSSVSSKD DADIVL
CBS12357_MALR_Chr16    SALITLPITR PLQPNGDSKD DTDIIL
CBS12357_MALR_Chr5     SALITLPITR PLQPNGDSKD DTDIIL
CBS12357_MALR_Chr13.1  SALITLPIAK PLKSGERFA- ------
CBS12357_MALR_Chr13.2  SSLVALPISK PLQL------ ------
S_uvarum_MALR_Chr5     SALITLPISR PLQSTNDSKD DVDIIL
S_uvarum_MALR_Chr16    RALITLPIAK PLQSSSSSKD DTDIIL
S_cerevisiae_MAL13     NALITLPISK PLQLDGYPKD NEDIDP
S_cerevisiae_MAL33     SALIDLPISR PLRLNDDSKD EDDIIP
S_cerevisiae_MAL63     SALIDLPISR PLRLNDDSKD DVDIIP
S.paradoxus            SALIDLPIPR PLRLSDDSKD DADIIP
S.mikatae              SALITLPISR PPKSSEDLRK R-----
```

Supplementary Figure 2: MalR protein sequence alignment across *Saccharomyces* species. Protein sequences were aligned using the Clustal Omega algorithm as implemented by Seaview. The suggested DNA binding domain, and the residues of the W307L and I243N substitutions are highlighted.

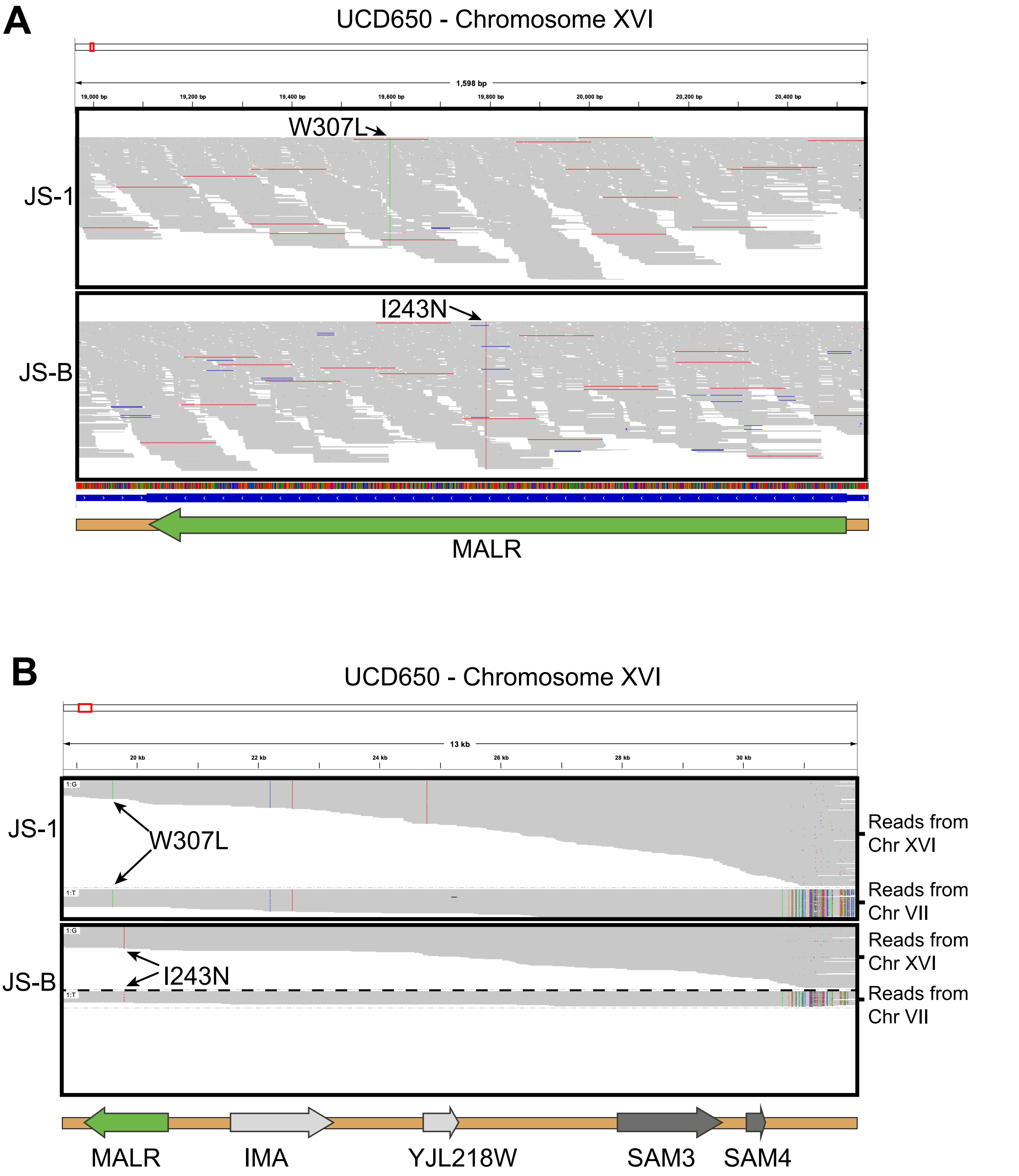

Supplementary Figure 3: Alignment of short and long reads show that the I243N and W307L mutations are homozygous at both *MALR*^{VII} and *MALR*^{XVI} in the evolved strains JS-1 and JS-B. (A) Short-read sequences aligned to the UCD650 genome at the *MALR*^{XVI} locus. Each grey horizontal line represents an aligned sequencing read. The green vertical line marked 'W307L' highlights that all reads from JS-1 contain the variant causing the W307L mutation. Similarly, the red vertical line marked 'I243N' shows that all reads from JS-B contain the variant causing the I243N mutation. (B) Long-read sequencing provides reads that span the 30-kb duplication and can be anchored to either chromosome XVI or chromosome VII. For both JS-1 and JS-B, all reads from both chromosome XVI and chromosome VII contain the respective variant, displayed similarly to panel (A).

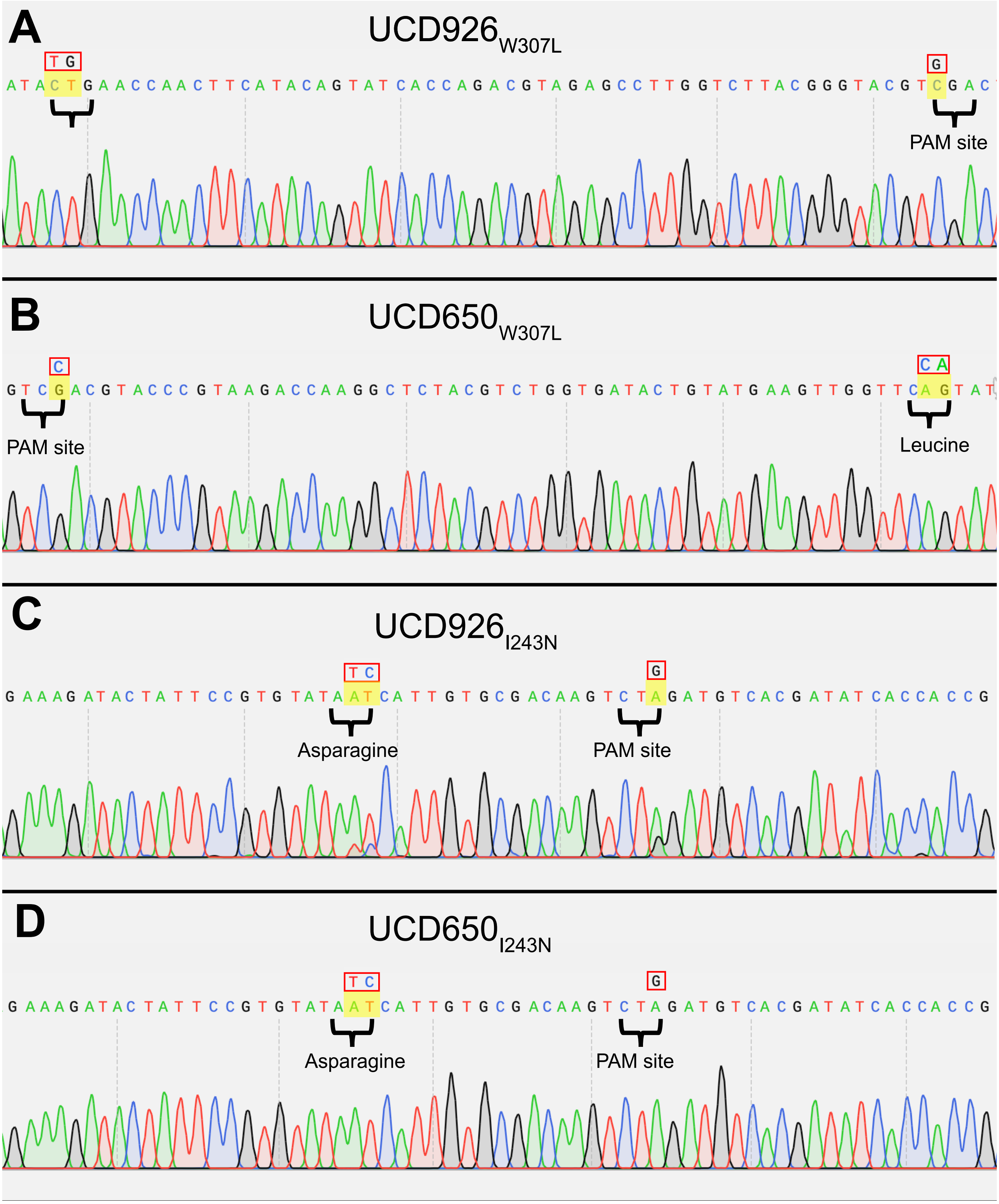

Supplementary Figure 4: Sanger sequencing chromatograms showing the four different single–amino acid substitutions introduced into the *MALR* genes of *S. eubayanus* strains UCD650 and UCD926 using CRISPR–Cas9–mediated genome editing.
Yellow highlighting indicates the edited nucleotide positions in the chromatograms, while red boxes above show the corresponding wild-type nucleotide sequences. Synonymous mutations introduced to disrupt the guide RNA NGG PAM site are also indicated, with the wild-type nucleotides shown above in red boxes.
(A) *MALR* W307L substitution in UCD926.
(B) *MALR* W307L substitution in UCD650. The sequence shown is the reverse strand.
(C) *MALR* I243N substitution in UCD926; mixed peaks at the edited nucleotides indicate incomplete editing of all MALR.
(D) *MALR* I243N substitution in UCD650.
